# Mutation type classification and pathogenicity assignment of sixteen missense variants located in the EGF-precursor homology domain of the LDLR

**DOI:** 10.1038/s41598-020-58734-9

**Published:** 2020-02-03

**Authors:** Unai Galicia-Garcia, Asier Benito-Vicente, Kepa B. Uribe, Shifa Jebari, Asier Larrea-Sebal, Rocio Alonso-Estrada, Joseba Aguilo-Arce, Helena Ostolaza, Lourdes Palacios, Cesar Martin

**Affiliations:** 1 Instituto Biofisika (UPV/EHU, CSIC), 48080 Bilbao, Spain; 2 Instituto Biofisika (UPV/EHU, CSIC) and Departamento de Bioquímica, Universidad del País Vasco, Apdo. 644, 48080 Bilbao, Spain; 3 Progenika Biopharma, a Grifols Company, Derio, Spain

**Keywords:** Dyslipidaemias, Dyslipidaemias

## Abstract

The primary genetic cause of familial hypercholesterolemia (FH) is related to mutations in the *LDLR* gene encoding the Low-density Lipoprotein Receptor. LDLR structure is organized in 5 different domains, including an EGF-precursor homology domain that plays a pivotal role in lipoprotein release and receptor recycling. Mutations in this domain constitute 51.7% of the total missense variants described in LDLR. The aim of the present work was to analyse how clinically significant variants in the EGF-precursor homology domain impact LDLR. The activity of sixteen *LDLR* variants was functionally characterized by determining LDLR expression by Western blot and LDLR expression, LDL binding capacity and uptake, and LDLR recycling activity by flow cytometry in transfected CHO-*ldl*A7 cells. Of the analysed variants, we found six non-pathogenic LDLR variants and ten pathogenic variants distributed as follow: three class 3 variants; four class 2 variants; and three class 5 variants. These results can be incorporated into clinical management of patients by helping guide the appropriate level of treatment intensity depending on the extent of loss of LDLR activity. This data can also contribute to cascade-screening for pathogenic FH variants.

## Introduction

Familial hypercholesterolemia (FH; MIM# 143890) is a common genetic disorder that leads to severely high low density lipoprotein cholesterol (LDL-C) levels from birth. If untreated, FH significantly increases cardiovascular risk^1,2^ and results in early onset of atherosclerosis which leads to increased risk of premature heart attack, stroke and death^3^. Among all the proteins implicated in the pathology of this disease, the *low density lipoprotein receptor (LDLR)* (MIM# 606945) is the most common genetic cause, and mutations within it are responsible of approximately 80–85% of FH cases^4^. To date, more than 2600 *LDLR* variants have been described (ClinVar database).

The *LDLR* gene is located on the short arm of chromosome 19 (19p13.1–13.3) with a length of approximately 45 kb encoding 18 exons and 17 introns. LDLR is a protein of 839 amino acids that is synthesized in the endoplasmic reticulum (ER), where it folds and is partially glycosylated. Next, LDLR is further glycosylated in the Golgi apparatus, rendering the mature protein^5^. The LDLR is organized in five functionally distinct domains: the N-terminal ligand-binding domain, the epidermal growth factor (EGF)-precursor homology domain, the O-linked sugars containing domain, the trans-membrane and the C-terminal cytosolic domain^6^.

Mutations in *LDLR* can impair LDLR activity at different levels and therefore are classified according to their phenotypic behaviour as: class 1 (no protein synthesis), class 2 (partial or complete retention of LDLR in the ER), class 3 (defective binding to apolipoprotein B (apoB), class 4 (defective endocytosis) and class 5 (diminished LDLR recycling capacity)^7,8^.

The physiologic activity of LDLR is to carry lipoproteins into cells, most commonly low density lipoprotein (LDL)^9^. Upon LDL binding to LDLR, the ligand-receptor complex internalizes through clathrin- mediated endocytosis. Cargo is then released by endosome acidification, a process that allows LDLR recycling back to the cell membrane while LDL is degraded in the lysosomes. Failure in cargo release results in lysosomal degradation of the LDL-LDLR complex^10^.

The EGF-precursor homology domain (411 amino acids in length) plays a pivotal role in lipoprotein release and receptor recycling processes. It consists of two EGF-like domains (EGF-A and EGF-B), six YWTD repeats that form a six-bladed β-propeller, and a third EGF-like repeat (EGF-C)^11^. Ligand release is an acid-dependent process, where due to endosome acidification, the LDLR conformation switches from an open (ligand-active) to a closed (ligand-inactive) conformation^12^. Although the mechanism underlying this conformational change is still unclear, it has been proposed that three histidine residues located at the interface between the fifth repetition of the ligand-binding domain (LR5) and the β-propeller act as pH sensors that allow the necessary flexing of the LDLR for subsequent conformational change^13^. It has also been proposed that, in endosomes, the β-propeller displaces the bounded lipoprotein ligand thereby acting as an alternative substrate for the ligand-binding domain^12^. Consequently, conformational change could be facilitated by interaction between the β-propeller and the main ligand-binding domain repeats (LR4 and LR5). In addition, it has been shown that the stability of LR5 decreases drastically due to the decreased pH and Ca^2+^ concentrations in the endosome thus triggering LR5 unfolding and consequently LDL release from the receptor^13^.

The development of new high throughput screening sequencing technologies allows detection of new variants in different populations continuously^14,15^, however, in order to provide an accurate genetic FH diagnosis, the LDLR variants must be functionally characterized to avoid misdiagnosis. To date, more than half of the reported missense mutations have not been functionally validated. Although co-segregation studies are very useful in assessing variant pathogenicity^16–18^ understanding how alterations disturb the function of the protein is necessary to develop personalized treatment.

**Figure 1:**
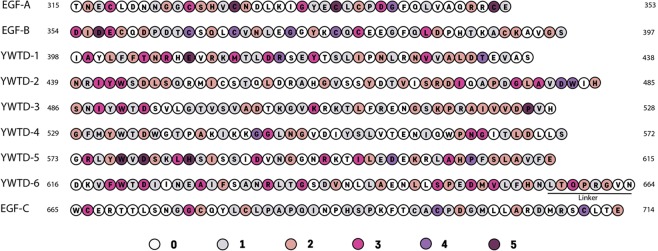
Location and frequency of LDLR variants within the EGF-precursor homology domain according to ClinVar database. Colours represent the number of described variants at a given amino acid.

To date, 1,108 missense variants localized in exons 7 to 14 (corresponding to the EGF-precursor homology domain) have been annotated in the ClinVar database (as of 10/12/2019), representing the 51.7% of the total missense variants described in the LDLR. The extremely high frequency of missense mutations occurring within the EGF-precursor homology domain is shown in Fig. 1, and the specific amino acid substitution at each position are indicated in supplementary Tables S1–S9 together with their clinical significance and review status. As shown in Fig. 1, there are variants in almost all the residues within the EGF-precursor homology domain, in some cases with as many as 5 variants for a given residue. This makes it difficult to assess the activity of all them *in vitro*; however the effort of characterizing as many as possible will provide useful information in order to understand both how these mutations can affect LDLR activity as well as structural information of the domain.

Given the high frequency of missense mutations occurring in the LDLR EGF-precursor homology domain and its relevant function in receptor structure maintenance and recycling, the aim of the present work was to analyse the impact on the LDLR activity of sixteen missense variants located in the EGF-precursor homology domain found in FH patients. Specifically, the activity of the LDLR p.(Ser326Cys), p.(Cys338Phe), p.(Cys368Tyr), p.(Gln378Pro), p.(Ala399Thr), p.(Thr413Met), p.(Asp492Asn), p.(Ser584Pro), p.(Ala606Ser), p.(Aps622Gly), p.(Arg633Cys), p.(His656Asn), p.(Thr659Asn), p.(Cys698Tyr), p.(asp700Gly) and p.(Asp707Tyr) variants was assessed by determining LDLR expression by Western blot and LDLR expression, LDL binding capacity and uptake and, LDLR recycling activity by flow cytometry in transfected CHO-*ldl*A7 cells. The results obtained here, showing ten pathogenic LDLR variants, can be translated into clinical management of patients by allowing clinicians to match treatment intensity to the extent of LDLR variant activity loss.

## Materials and Methods

### LDLR variant selection

In order to select variants in the EGF-precursor homology domain of the LDLR that could have an impact in the activity of the receptor, we used the ClinVar database (https://clinvarminer.genetics.utah.edu) and selected the following ones: p.(Ser326Cys), p.(Cys338Phe), p.(Cys368Tyr), p.(Gln378Pro), p.(Ala399Thr), p.(Thr413Met), p.(Asp492Asn), p.(Ser584Pro), p.(Ala606Ser), p.(Aps622Gly), p.(Arg633Cys), p.(His656Asn), p.(Thr659Asn), p.(Cys698Tyr), p.(asp700Gly) and p.(Asp707Tyr). These *LDLR* variants were selected because they have been described in FH patients, their clinical interpretation was not previously assessed and we did not find any study that had functionally characterized them. All these variants have also been found by the LIPOchip® platform and/or by the SEQPRO LIPO S® platform from Progenika Biopharma (Derio, Spain), both having the CE mark. Description of the studied variants, conservation and *in silico* predictions is shown in Table 1.

**Table 1:** Description of LDLR variants, conservation and *in silico* predictions.

Genetic name	HGVS Nomenclature	Conservation nt	Conservation AA	Grantham distance	Align GVGD	SIFT	PolyPhen-2	MutationTaster 2
c.977 C > G	p.(Ser326Cys)	1.00	0.97	112	C65	Not tolerated	probably damaging (1)	Disease causing (prob: 1)
c.1013 G > T	p.(Cys338Phe)	1.00	1.00	205	C65	Not tolerated	probably damaging (1)	Disease causing (prob: 1)
c.1103 G > A	p.(Cys368Tyr)	1.00	1.00	194	C65	Not tolerated	probably damaging (1)	Disease causing (prob: 1)
c.1133 A > C	p.(Gln378Pro)	1.00	0.24	76	C25	Not tolerated	benign (0.155)	Disease causing (prob: 0.994)
c.1195 G > A	p.(Ala399Thr)	0.86	0.94	58	C0	Not tolerated	probably damaging (0.991)	Disease causing (prob: 0.996)
c.1238 C > T	p.(Thr413Met)	0.94	0.89	81	C15	Not tolerated	probably damaging (1)	Disease causing (prob: 1)
c.1474 G > A	p.(Asp492Asn)	1.00	1.00	23	C0	Not tolerated	probably damaging (1)	Disease causing (prob: 1)
c.1750T > C	p.(Ser584Pro)	0.85	0.85	74	C0	Not tolerated	possibly damaging (0.953)	Disease causing (prob: 1)
c.1816G > T	p.(Ala606Ser)	0.87	0.87	99	C0	Tolerated	benign (0.067)	Disease causing (prob: 0.999)
c.1865A > G	p.(Asp622Gly)	1.00	1.00	94	C65	Not tolerated	probably damaging (1)	Disease causing (prob: 1)
c.1897C > T	p.(Arg633Cys)	0.92	0.95	180	C55	Not tolerated	probably damaging (0.987)	Disease causing (prob: 1)
c.1966C > A	p.(His656Asn)	1.00	1.00	68	C65	Not tolerated	possibly damaging (0.597)	Disease causing (prob: 1)
c.1976C > A	p.(Thr659Asn)	0.72	0.69	65	C0	Tolerated	benign (0.003)	Polymorphism (prob: 0.997)
c.2093 G > A	p.(Cys698Tyr)	1.00	1.00	194	C65	Not tolerated	probably damaging (0.998)	Disease causing (prob: 1)
c.2099 A > G	p.(Asp700Gly)	1.00	1.00	94	C0	Tolerated	probably damaging (0.999)	Disease causing (prob: 1)
c.2119 G > T	p.(Asp707Tyr)	1.00	0.97	160	C65	Not tolerated	probably damaging (1)	Disease causing (prob: 1)

### *In silico* predicted effect of molecular event on LDLR

To predict the possible impact of the studied variants, four different software programs were used: Align GVGD (http://agvgd.iarc.fr)^19^, PolyPhen-2 (http://genetics.bwh.harvard.edu/pph2)^20^, SIFT (http://sift.jcvi.org)^21^ and MutationTaster2 (http://www.mutationtaster.org)^22^. GVGD and SIFT analysis for amino acid substitutions were performed by comparing amino acid substitutions in human LDLR peptide sequence (P01130) against LDLR amino acid sequences from 9 species: Pan troglodytes (XP_001167447.1), Macaca mulatta (NP_001028078.1), Mus musculus (P35951.1), Rattus norvegicus (P35952.1), Oryctolagus cuniculus (P20063.1), Sus scrofa (Q28832.2), Equus caballus (XP_001490793.2), Xenopus laevis (AAI70345.1) and Danio rerio (NP_001025454.1). The dataset HumDiv was used for testing with PolyPhen-2 prediction software (version 2.2.2). MutationTaster2 employs a Bayes classifier to eventually predict the disease potential of an alteration. The Bayes classifier is regularly updated, i.e. predictions might in some cases change over time. The prediction programs usually give a prediction probability value based on its specific model. In the MutationTaster2 and PolyPhen-2, the probability value (in brackets) is the probability of the prediction, i.e. a value close to 1 indicates a high ‘security’ of the prediction. In SIFT, the score ranges from 0 to 1. The amino acid substitution is predicted deleterious if the score is <0.05, and tolerated if the score is >0.05. The GVGD programs score goes from 0 (no pathogenic) to 65 (highly predicted as pathogenic).

### Conservation analysis

Conservation analysis among species for nucleotide and amino acid was carried out using data from the University of California, Santa Cruz (UCSC) genome browser (http://genome.ucsc.edu), based on multiz aligments of 46 vertebrate specie sequences^23^ and expressed as the ratio of sequences harbouring the human residue divided by the total of available sequences.

### Construction of LDLR variants carrying plasmids

Plasmids containing the LDLR variants were designed by Innoprot (Derio, Spain). Using the QuickChange Lightning mutagenesis kit (Agilent). The mammalian expression vector pcDNA3 was used to introduce the variants into the human LDLR cDNA (NM_000527.4) under control of a SV40 promoter by oligonucleotide site-directed mutagenesis and according to manufacturer´s instructions. In order to generate the plasmids carrying the LDLR variants, oligonucleotides were synthesized *in vitro* and subcloned using SacII and EcoRI restriction enzymes. The presence of the desired nucleotide alteration was confirmed by PCR and restriction enzyme digestion of the appropriate fragments, while direct sequence analysis was used to verify the integrity of the remaining LDLR cDNA sequence of the construct.

### CHO-*ldl*A7 cell culture and transfection

CHO-*ldl*A7 cells not expressing LDLR (kindly provided by M. Krieger, MIT, MA, USA) were maintained in Ham’s F-12 medium containing 10% FBS, 2 mM L-glutamine and antibiotics (100 units/mL penicillin; 100 µg/mL streptomycin). Cells were grown into 6- or 24- well culture plates and later on transfected with Lipofectamine® LTX-PlusTM Reagent (Invitrogen) following manufacturer’s indication when an 80% of confluence was reached. 48 hours after transfection LDLR functionality was assessed.

### Immunodetection of LDLR

Cells were lysed in ice cold 50 mM Tris-HCl buffer containing 125 mM NaCl, 1% Nonidet P-40, 5.3 mM NaF, 1.5 mM NaP, 1 mM orthovanadate, 1 mg/mL complete EDTA-free protease inhibitor cocktail (Roche), 0.25 mg/mL Pefabloc, 4-(2-aminoethyl)-benzenesulfonyl fluoride hydrochloride (AEBSF; Roche), pH 7.5. Cells were rotated at 4 °C for an hour and centrifuged at 12,000 × g during 15 minutes to remove insoluble material. Protein fractionation was carried out by electrophoresis on non-reducing 8.5% SDS-PAGE for semi-quantitative inmunobloting. Antibodies used were the following: rabbit polyclonal anti-LDLr antibody (1:500) (Progen Biotechnik GimbH, Heidelberg, Germany), anti-GAPDH antibody (1:1000) (Nordic Biosite, Little Chalfont, UK). Primary antibodies were incubated overnight at 4 °C, while secondary antibody was incubated at room temperature for 1 hour. Signals were developed using SuperSignal West Dura Extended Substrate (Pierce Biotechnology, Rockford, IL, USA) in a ChemiDoc XRS (Bio-Rad, Hercules, CA, USA). Band intensity quantification was performed using NHI ImageJ software (https://rsbweb.nih.gov/ij/) and levels of protein of interest were corrected to GAPDH.

### LDL isolation and labelling

Blood plasma was collected from healthy individuals after 30 min centrifugation at 2,000 × g at 4 °C. In order to isolate LDL (1.019–1.050 g/mL) by a sequential ultracentrifugation, plasma density was adjusted to 1.21 g/mL adding KBr. Afterwards, a second ice-cold PBS buffer was slowly added at the top of the solution generating a two phase gradient. Ultracentrifugation was carried out in a SW28.1 rotor (Beckman Coulter, USA) at 27,000 rpm for 22 h at 4 °C. Then, the band corresponding to LDL was collected and stored at 4 °C. LDL was used within 2–3 days after purification. LDL was fluorescently labelled with fluorescein isothiocyanate (FITC) as described previously^24^. Briefly, LDL was incubated with 10 µL/mL FITC in 0.1 M NaHCO_3_ (pH 9.0) at room temperature under slight agitation for 2 h. Once incubation was completed, the non-bounded FITC was removed by washing the lipoprotein solution in a previously PBS EDTA-free balanced Sephadex G-25 column. Protein concentration was determined in all fractions using BSA as standard (Pierce BCA protein assay, Pierce). This study was approved by the Research Ethics Committee of the University of the Basque Country (Comité de Ética en la investigación y la práctica docente de la Universidad del País Vasco/Euskal Herriko Unibertsitatea; CEIAB/186/2014/MARTÍN PLÁGARO). Methods were carried out according to the approved guidelines. All participants signed the written informed consent. All experiments were carried out according to relevant guidelines and regulations.

### Analysis of LDLR expression by FACS

LDLR expression at the cell membrane was assessed in a FACScalibur using Mouse anti-human-LDLR (1:100; 2.5 mg/L; Progen Biotechnik GmbH) and Alexa Fluor 488-conjugated goat anti-mouse IgG (1:100; Molecular Probes) primary and secondary antibodies, respectively. Inmunostaining was performed as previously described^25^. Each sample was performed in triplicate and 10,000 events were acquired for data analysis.

### Analysis of LDLR activity by FACS

Cells were transfected as described above and, 48 h after transfection, FITC-LDL (20 µg/mL) was added to the cell culture medium. Cells were incubated during 2 h at 4 °C or 4 h at 37 °C in order to determine LDL binding and uptake, respectively. Then, cells were washed out with PBS-1% BSA, fixed in 4% paraformaldehyde for 10 minutes at room temperature and rinsed again to eliminate the surplus fixative. Trypan blue solution (0.2% final concentration, Sigma-Aldrich, Steinheim, Germany) was added to the samples to determine LDL uptake, allowing the quenching of the extracellular FITC signal coming from the non-internalized LDL–LDLR complexes. Geometric mean fluorescence intensity (GMFI) of each sample was determined in a FACScalibur Flow cytometer following the manufacturer´s instructions. GMFI of 10,000 events was obtained for each sample and every assay determination was performed at least three times.

### LDL–LDLR binding at different pH

CHO-*ldl*A7 cells were transfected with plasmids carrying the *LDLR* mutations as described before, and then incubated with 20 μg/ml of LDL–FITC for 30 min in a 0.4 M sucrose supplemented Ham’s medium at different pH. Then, cells were washed three times to remove the unbound LDL, fixed with 4% paraformaldehyde for 10 min, and washed again with Ham’s medium at its corresponding pH in each case. Bound LDL–FITC was determined as described before.

### Statistical analysis

All measurements were performed at least 3 times, with n = 3 unless otherwise specified and results represent the mean ± S.D. The differences between LDLR variants and wt LDLR were tested by a two-tailed Student´s t-test with a significance level of 0.05 considered to be statistically significant.

## Results

### Characteristics of the *LDLR* studied variants, conservation and *in silico* predictions

The amino acid conservation and *in silico* predictions, determined by different software packages, are shown in Table 1. Most of the variants are classified as damaging or possible damaging, only the p.(Thr659Asn) change is classified as non-pathogenic by the 4 prediction programs. The *in silico* predictions showed different predictions, even more, for several variants its prediction among programs is the opposite. The inconsistency among algorithms emphasises the importance of performing a functional characterization of the variants.

### Expression of LDLR variants in CHO-*ldl*A7 cells

**Figure 2:**
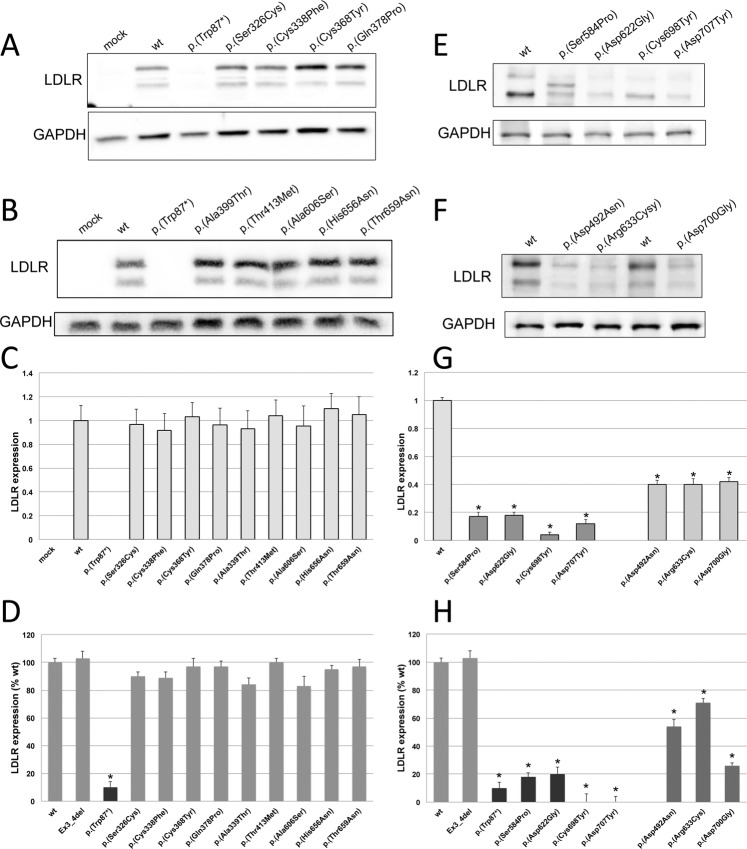
Expression of wt and sixteen LDLR variants in CHO-*ldlA7* transfected cells. Expression of LDLR was determined 48 h post-transfection with the plasmids carrying the different LDLR variants by Western blot and flow cytometry. (**A,B,E,F)** Western blot analysis of LDLR expression. (**C,G**) Quantification of the intensities of the bands obtained by Western blot by densitometry and, (**D,H)** LDLR expression determined by flow cytometry. A representative blot is shown in panel A,B,E and F. C and G represent the mean of band quantification of three independent Western blots. The values in D and H represent the mean of triplicate determinations (n = 3); error bars represent ± SD. *P < 0.001 compared to wt using a Student’s t-test.

Expression of the sixteen LDLR variants was analysed by Western blot and by flow cytometry in CHO-*ldl*A7 transfected cells as described in Materials and Methods. Two variants were used as internal-method controls, p.(Trp87)* (a null allele mutant) and, Ex3_4del LDLR variant that is expressed at similar extent than wt LDLR but it is a class 3 variant with 100% impaired binding activity^24^. According to the obtained results, expression of the assessed variants can be classified into three categories, those that are expressed similarly than wt, those with lower expression than wt, and finally those who are not expressed. As shown in Fig. 2A,B, expression of p.(Ser326Cys), p.(Cys338Phe), p.(Cys368Tyr), p.(Gln378Pro), p.(Ala399Thr), p.(Thr413Met), p.(Ala606Ser), p.(His656Asn) and p.(Thr659Asn) LDLR variants is similar than wt LDLR expression determined 48 h post-transfection by Western blot, as confirmed by quantification of the relative band intensity of mature LDLR protein expressed as the ratio between the 130 kDa band to that of GADPH by densitometric analysis (Fig. 2C). Similar results were obtained when LDLR expression was determined by flow cytometry (Fig. 2D). On the other hand, p.(Asp492Asn), p.(Arg633Cys) and p.(Asp700Gly) are among the LDLR variants showing diminished LDLR expression, as determined by both Western blot and flow cytometry (Fig. 2E,G,H). Finally, expression of mature p.(Ser584Pro), p.(Asp622Gly), p.(Cys698Tyr) and p.(Asp707Tyr) was not detected by Western blot (Fig. 2F,G) and consequently, flow cytometry assays showed no expression at cell surface of these three variants (Fig. 2H).

### LDLR activity (LDL binding and uptake) of LDLR variants determined by flow cytometry

**Figure 3:**
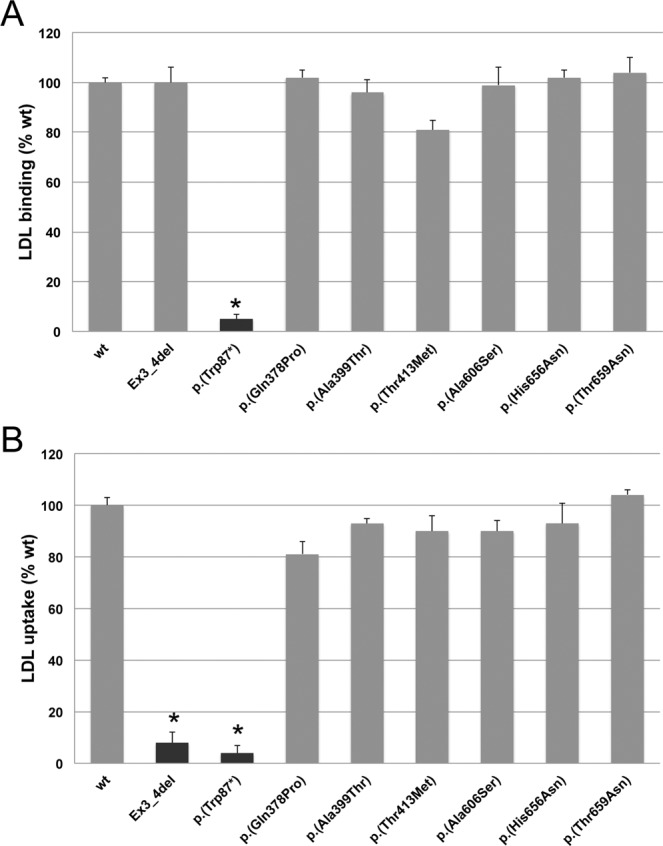
LDLR activity of wt and p.(Gln378Pro), p.(Ala399Thr), p.(Thr413Met), p.(His656Asn), p.(Thr659Asn) and p.(Ala606Ser) LDLR variants. (**A**) LDL-LDLR binding and (**B**) FITC-LDL uptake activity. Assays were performed as described in Materials and Methods. Data show the mean of three independent experiments; error bars represent ± SD. *P < 0.001 compared to wt using a Student’s t-test.

**Figure 4:**
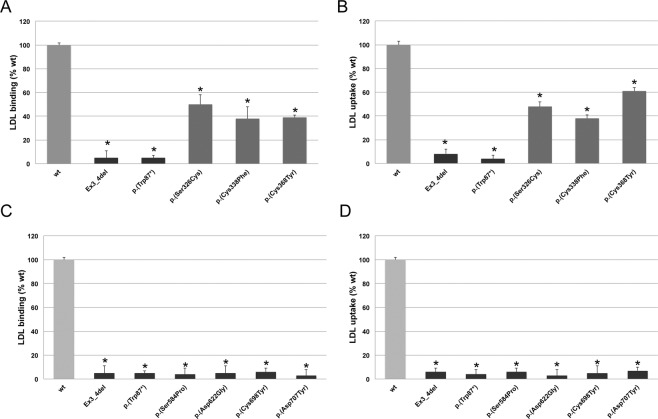
LDLR activity of wt and p.(Ser326Cys), p.(Cys338Phe), p.(Cys368Tyr), p.(Ser584Pro), p.(Asp622Gly), p.(Cys698Tyr) and p.(Asp707Tyr) LDLR variants. (**A,C)** LDL- LDLR binding and (**B,D)** FITC-LDL uptake activity. Assays were performed as described in Materials and Methods. Data show the mean of three independent experiments; error bars represent ± SD. *P < 0.001 compared to wt using a Student’s t-test.

Activity of the LDLR variants was assessed in CHO-*ldlA*7 cells transfected cells as described in Materials and Methods. LDL binding and uptake was determined by FACS as previously described^24^. As shown in Fig. 3A,B, both LDL-LDLR binding activity and LDL uptake of p.(Gln378Pro), p.(Ala399Thr), p.(Thr413Met), p.(Ala606Ser), p.(His656Asn) and p.(Thr659Asn) LDLR variants were similar than wt LDLR. On the other hand and as shown in Fig. 4A,B, p.(Ser326Cys), p.(Cys338Phe), p.(Cys368Tyr) LDLR variants, which have a similar expression to wt LDLR as determined in the previous section, showed statistically significant reduced LDL-LDLR binding activity (wt: 100 ± 2; p.(Ser326Cys): 50 ± 8, p.(Cys338Phe): 38 ± 10, p.(Cys368Tyr): 39 ± 2) and LDL uptake (wt: 100 ± 3; p.(Ser326Cys): 48 ± 4, p.(Cys338Phe): 38 ± 3, p.(Cys368Tyr): 61 ± 3). On the other hand, and expected due to the LDLR expression assay, p.(Ser584Pro), p.(Asp622Gly), p.(Cys698Tyr) and p.(Asp707Tyr) showed residual LDLR activity (Fig. 4C,D).

**Figure 5:**
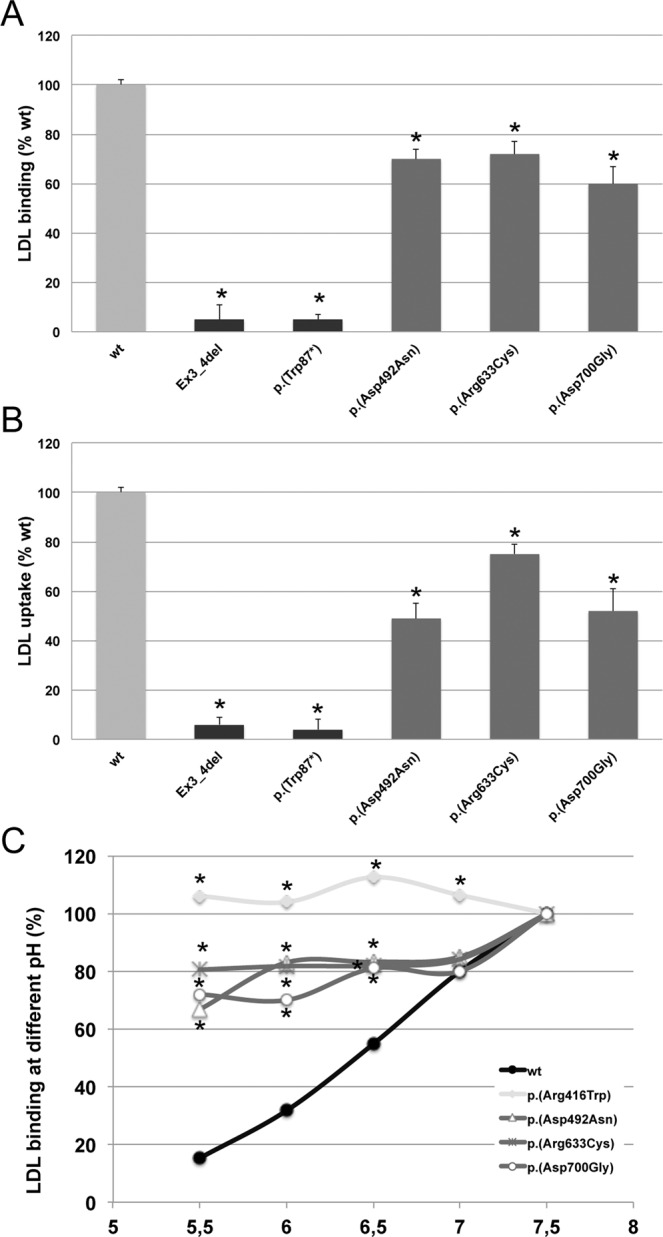
LDLR activity of wt and p.(Asp492Asn), p.(Arg633Cys), and p.(Asp700Gly) LDLR variants. (**A**) LDL- LDLR binding, (**B**) FITC-LDL uptake activity and (**C**) LDL binding at different pH. Assays were performed as described in Materials and Methods. Data show the mean of three independent experiments; error bars represent ± SD. *P < 0.001 compared to wt using a Student’s t-test.

Finally, and according to the LDLR expression results, p.(Asp492Asn), p.(Arg633Cys) and p.(Asp700Gly) LDL binding activity was significantly diminished compared to wt LDLR (LDL binding: wt: 100 ± 2; p.(Asp492Asn): 70 ± 4; p.(Arg633Cys): 72 ± 5; and p.(Asp700Gly): 60 ± 7 (Fig. 5A). In addition, and corroborating these results, LDL uptake was also significantly diminished in comparison to the uptake values for wt: 100 ± 2; p.(Asp492Asn): 49 ± 6; p.(Arg633Cys): 75 ± 4; and p.(Asp700Gly): 52 ± 9 (Fig. 5B).

### p.(Asp492Asn), p.(Arg633Cys) and p.(Asp700Gly) LDLR variants affect negatively to receptor recycling

The reduced expression (30–70% compared to wt LDLR) and LDLR activity (≈60–70% LDL binding and ≈45–75% uptake compared to wt LDLR) together with the absence of an accumulation of the non-processed LDLR in p.(Asp492Asn), p.(Arg633Cys) and p.(Asp700Gly) LDLR variants (Figs. 2E,F,H and 5A,B, respectively) is an indicative of a possible defect in LDLR recycling. Accordingly, we next determined LDL binding capacity of the LDLR variants at different pH (5.5–7.4). The rationale of this assay is to mimic the acid-dependent mechanism of lipoprotein release in the endosomal compartment. Diminished LDL binding at pH 5.5 compared to binding at pH 7.4 would resemble the dissociation of the LDL-LDLR complex occurring upon endosomal acidification, which allows the required structural change for the LDLR to be recycled to the membrane. The opposite result, constant LDL-LDLR binding rate, would indicate that acidification does not promote complex dissociation and LDLR would be driven, together with LDL, to lysosomes for degradation. Accordingly, we studied LDL–LDLR binding at different pH to mimic LDL release induced by pH acidification occurring at endosomes. As shown in Fig. 5C, LDL binding was pH dependent for wt, with LDL binding at pH 5.5 being 80% less efficient compared with that at pH 7.5. In this assay we included p.(Arg416Trp) LDLR variant as an internal-control of the method because it has been previously classified as a class 5 LDLR variant^26^. As expected, LDL binding to p.(Arg416Trp) LDLR variant was not affected by acidification (Fig. 5C). Similarly to the results obtained with the p.(Arg416Trp) LDLR control, in p.(Asp492Asn), p.(Arg633Cys) and p.(Asp700Gly) LDLR variants, LDL release at acidic pH was not as efficient as in wt. Binding in p.(Asp492Asn) variant at pH 5.5 diminished 37% compared with binding at pH 7.5, binding of LDL to p.(Arg633Cys) at pH 5.5 was 42% less efficient than at pH 7.5 and binding of p.(Asp700Gly) LDLR variant was diminished 36% compared to pH 7.5 (Fig. 5C).

## Discussion

A comprehensive assessment of FH patients that combines clinical and molecular data from patients with functional characterization and classification of LDLR variants can elucidate the spectrum of FH phenotypes and provide insight into the development of targeted treatments^27^. Therefore, in this work, in order to align molecular characterization of LDLR variants with clinical severity, we have functionally characterized and classified sixteen LDLR variants located in the EGF-precursor homology domain of the LDLR. These variants have been previously described in FH patients but without prior clinical interpretation or functional characterization. Due to the role of EGF-precursor homology domain in maintaining the correct folding at cell surface (supported by YWTD repeats) and as a pH sensor that allows receptor recycling, the majority of functional-defective variants are class 2 (partial or complete retention of LDLR in the ER), and class 5 (diminished LDLR recycling capacity) mutations^7,28^. These findings highlight the importance of characterizing and classifying *LDLR* variants in order to provide and accurate diagnosis and treatment of FH. Although laborious and time consuming, a correct assessment of pathogenicity and classification of a large amount of variants can not just rely on *in silico* characterization due to the lack of a robust computational software able to accurately predict the functional effects of variants. Current software compares amino acid or nucleotide conservation among species and basic biochemical features of the substituted amino acid. However, it does not consider other parameters such as interaction of the substituted amino acid with its surrounding residues and how such interactions can modify protein structure or activity. Among the sixteen variants selected in this work, p.(Thr659Asn) was the only variant correctly predicted as non-pathogenic by the four softwares tested, which was consistent with our *in vitro* characterization of that variant. For the remaining fifteen LDLR variants, *in silico* analysis showed contradictory interpretations that do not allow drawing any conclusion regarding protein activity. This discrepancy occurs with both non-pathogenic variants and pathogenic variants. In particular, of the non-pathogenic variants identified by *in vitro* experiments, four of the six variants analysed (p.(Gln378Pro), p.(Ala399Thr), p.(Thr413Met) and p.(His656Asn)) were considered to be pathogenic by at least two predictors.

Very interestingly, among the sixteen characterized LDLR variants, in six of them the amino acid alteration did not cause loss of receptor activity thus constituting neutral alterations. Although they were identified in FH patients, p.(Gln378Pro), p.(Ala399Thr), p.(Thr413Met), p.(Ala606Ser), p.(His656Asn) and p.(Thr659Asn) LDLR variants were found to be non-pathogenic thereby indicating that the variants do not account for the clinical presentation. Consequently, the cause of FH in patients carrying these variants should be further investigated in order to identify a cause. Of course, cascade screening to find FH relatives based on these variants is recommended.

We also identified four class 2a LDLR variants in which the amino acid substitution leads to a complete retention of the immature protein at the ER. These identified class 2a variants are p.(Ser584Pro), p.(Asp622Gly), p.(Cys698Tyr) and p.(Asp707Tyr), all showing a residual LDLR activity. A detailed analysis of the impact of the substituted amino acids in the protein structure provides information about the molecular mechanisms by which these variants are pathogenic.

In the case of p.(Ser584Pro), interestingly, in addition to the band corresponding to the non-mature LDLR, we detected by Western blot a band of an intermediate molecular weight between mature and immature LDLR (Fig. 2F). As determined by flow cytometry, there is no detection of LDLR at cellular surface, indicating that the LDLR-form corresponding to that band remains intracellularly retained. Based on the position of the replaced amino acid, there is not any disturbed N-linked site glycosylation, but an O-linked glycosylation site could have been lost. Additionally, since the Western blot was performed under non-reducing conditions, the intermediate band could correspond to a non-correctly folded protein with different secondary or tertiary structure compared to wt LDLR. In agreement with the latter, in the wt LDLR, there is an interaction between the backbones of Ser584 and Asp579, with Asp579 being one of the conserved amino acids within the fifth YWTF motif. As mentioned before, the six YWTD motifs of the β-propeller are packed as six four-stranded β-sheets (“blades”) that maintain the domain structure, which is determinant for a correct folding of β-propeller^9^. Moreover, the interaction between Ser584 and Asp579 allows a loop formation that accommodates a lysine, which in turn interacts with the ligand-binding domain at its closed conformation. Substitution of Ser584 by proline (an amino acid with lower flexibility) may destabilize the highly conserved structure of the YWTD motif causing ER retention.

Regarding the p.(Asp622Gly) LDLR variant, the amino acid substitution is located in the sixth YWTD, in a highly conserved region of the β-propeller. Asp622 interacts with the backbones of Ile623 and Ala627 allowing a loop formation that could be destabilized by a glycine substitution. Moreover, YWTD tetrapeptide repeats are essential for the correct β-propeller structure maintenance and proper receptor folding. Indeed, modifications in these sequences have previously been described as disease causing^25,29^ suggesting that Asp622 modification could not be well tolerated.

The p.(Cys698Tyr) and p.(Asp707Tyr) LDLR variants affect the EGF-C domain and are also retained at ER being class 2 variants. Substitution of Cys698 by a tyrosine leads to a loss of a disulfide bridge between Cys698 and Cys711 probably causing an incorrect folding of the protein resulting in ER retention. Unfortunately, the effect on the LDLR structure caused by the substitution of Asp707 by a tyrosine is not clear because the crystal structure surrounding that position is not well resolved.

On the other hand, we have found three class 3 variants: p.(Ser326Cys), p.(Cys338Phe) and p.(Cys368Tyr) LDLR variants. They showed similar expression to wt LDLR but demonstrated deficient LDL binding. In the wt LDLR, Ser326 backbone interacts with Asn322 and Val328 and its side chain interacts with Arg351. Substitution of Ser326 by a cysteine results in loss of interaction with Arg351 and more importantly, the location of two adjacent cysteines in a position involved in a disulfide bridge formation may disrupt the correct folding of the domain and negatively affect the ligand-binding domain structure. Similarly, the substitution of Cys338 by a phenylalanine causes a disulfide bridge disruption naturally occurring in wt between Cys325 and Cys338. It has been previously described that cysteine substitution at these two positions causes misfolding of EGF-A domain^30^ that can affect ligand-binding domain conformation. The Cys368 is located in the EGF-B shortly before the β-propeller and forms a disulfide bridge with the side chain of Cy358 and its backbone interacts with the backbone of Cys364. These interactions favour the generation of a loop that is organized around a calcium ion. Replacement of Cys368 by a tyrosine disrupts the disulfide bridge thus causing LDLR misfolding.

Finally, we have found three class 5 variants: p.(Asp492Asn), p.(Arg633Cys) and p.(Asp700Gly). Asp492 is located in the fourth repetition of the YWTD motif of the β-propeller. The interactions of Asp492 with the tryptophan located in the YWTD repeat have been described above for the p.(Asp622Gly) and p.(Asp707Tyr) class 2a LDLR variants. In this case, the substitution of Asp492 by asparagine leads to a class 5 LDLR variant probably because the substitute amino acid is relatively similar. In this way, although the protein structure is not affected in LDLR expression or LDL binding and uptake activities, the alteration introduced by the asparagine is enough to cause a defect in recycling of the protein. The p.(Arg633Cys) LDLR variant is located at the end of the β-propeller. In the wt protein, Arg633 interacts with Gln615 through its side chain and with its primary chain with Gln632 side chain. In addition, due to the β-propeller three-dimensional structure, Arg633 interacts with Ser665, an amino acid located between EGF-B and the β-propeller. Substitution of Arg633 by a cysteine completely disrupts the interactions with Gln615 and Ser365. Finally, Asp700 located in the EGF-C domain, establishes a hydrogen bond with Thr510, which is located in the β-propeller. This interaction seems to be necessary for a correct LDLR recycling because when Asp700 is replaced by a glycine, this interaction is lost and the p.(Asp700Gly) LDLR variant fails to undergo a correct recycling.

Although the FH clinical phenotype is highly variable, it has been suggested that depending on the class mutation, the effects of statin therapy can be affected^26,31,32^. Interestingly, although a similar lipid phenotype has been found in heterozygous patients carrying Class 2 and Class 5 variants, statin treatment is more effective at lipid level reduction in subjects with Class 2 LDLR variants than subjects with Class 5 LDLR variants^26^. These results highlight the importance of classification of LDLR variants in order to improve the efficacy or safety of therapy.

In conclusion, the data obtained in this work are complementary to clinical data in terms of both determining the extent of protein function impairment and variant classification. Integrating this information with clinical data improves patient clinical management in terms of accuracy of a definite diagnosis and advances in personalized medical care with the ultimate aim of tailoring therapy for maximal patient response.

## Supplementary information

Supplementary Material.

## Data Availability

All data generated or analysed during this study are included in this published article (and its Supplementary Information files).
