# Supplementary material for: Mutation type classification and pathogenicity assignment of sixteen missense variants located in the EGF-precursor homology domain of the LDLR
Source: Sci Rep. 2020 Feb 3;10:1727. doi: 10.1038/s41598-020-58734-9 (PMC6997160; doi:10.1038/s41598-020-58734-9)
Supplement: Supplementary file 1 — Supplementary Material. [file 41598_2020_58734_MOESM1_ESM.pdf]

Supplementary Information

Mutation type classification and pathogenicity assignment of sixteen missense variants located in the EGF-precursor homology domain of the LDLR

Unai Galicia-Garcia[1#], Asier Benito-Vicente[2#], Kepa B. Uribe[2], Shifa Jebari[2], Asier Larrea-Sebal[2], Rocio Alonso-Estrada[1], Joseba Aguilo-Arce[2], Helena Ostolaza[2], Lourdes Palacios[3], Cesar Martin[2*].

[1]Instituto Biofisika (UPV/EHU, CSIC), 48080 Bilbao, Spain.

[2]Instituto Biofisika (UPV/EHU, CSIC) and Departamento de Bioquímica, Universidad del País Vasco, Apdo. 644, 48080 Bilbao, Spain.

[3]Progenika Biopharma, a Grifols Company, Derio, Spain.

* Corresponding Author E-mail: cesar.martin@ehu.eus. Tel. +34-94-601.80.52

[#] These authors have contributed equally to this work.

Table S1: LDLR variants within the EGF-A domain annotated at ClinVar (10/12/2019).

EGF-A	Clinical significance	Review status
c.947A>C (p.Asn316Thr)	Likely pathogenic	criteria provided, multiple submitters, no conflicts
c.947A>G (p.Asn316Ser)	Likely pathogenic	criteria provided, single submitter
c.949G>A (p.Glu317Lys)	Pathogenic/Likely pathogenic	criteria provided, multiple submitters, no conflicts
c.952T>C (p.Cys318Arg)	Pathogenic/Likely pathogenic	criteria provided, multiple submitters, no conflicts
c.953G>T (p.Cys318Phe)	Likely pathogenic	criteria provided, multiple submitters, no conflicts
c.953G>A (p.Cys318Tyr)	Likely pathogenic	criteria provided, multiple submitters, no conflicts
c.965A>T (p.Asn322Ile)	Pathogenic/Likely pathogenic	criteria provided, multiple submitters, no conflicts
**c.967G>T (p.Gly323Cys)**	Pathogenic	criteria provided, single submitter
c.967G>A (p.Gly323Ser)	Likely pathogenic	criteria provided, multiple submitters, no conflicts
c.970G>A (p.Gly324Ser)	Conflicting interpretations of pathogenicity	criteria provided, conflicting interpretations
c.973T>C (p.Cys325Arg)	Likely pathogenic	criteria provided, single submitter
c.974G>T (p.Cys325Phe)	Likely pathogenic	criteria provided, single submitter
c.974G>A (p.Cys325Tyr)	Likely pathogenic	criteria provided, multiple submitters, no conflicts
c.977C>T (p.Ser326Phe)	Likely pathogenic	criteria provided, single submitter
c.977C>G (p.Ser326Cys)	Likely pathogenic	criteria provided, multiple submitters, no conflicts
c.979C>T (p.His327Tyr)	Conflicting interpretations of pathogenicity	criteria provided, conflicting interpretations
c.981C>A (p.His327Gln)	Likely pathogenic	criteria provided, single submitter
c.982G>A (p.Val328Ile)	Uncertain significance	criteria provided, single submitter
c.985T>C (p.Cys329Arg)	Likely pathogenic	criteria provided, single submitter
c.985T>G (p.Cys329Gly)	Pathogenic/Likely pathogenic	criteria provided, multiple submitters, no conflicts
c.986G>T (p.Cys329Phe)	Likely pathogenic	criteria provided, multiple submitters, no conflicts
c.986G>A (p.Cys329Tyr)	Conflicting interpretations of pathogenicity	criteria provided, conflicting interpretations
c.987C>G (p.Cys329Trp)	Likely pathogenic	criteria provided, single submitter
c.988A>C (p.Asn330His)	not provided	no assertion provided
c.990T>A (p.Asn330Lys)	Likely benign	criteria provided, single submitter
c.1003G>A (p.Gly335Ser)	Conflicting interpretations of pathogenicity	criteria provided, conflicting interpretations
c.1004G>T (p.Gly335Val)	Likely pathogenic	criteria provided, multiple submitters, no conflicts
c.1004G>A (p.Gly335Asp)	Likely pathogenic	criteria provided, single submitter
c.1007A>G (p.Tyr336Cys)	Pathogenic/Likely pathogenic	criteria provided, multiple submitters, no conflicts
c.1009G>A (p.Glu337Lys)	Uncertain significance	criteria provided, single submitter
c.1012T>G (p.Cys338Gly)	Likely pathogenic	criteria provided, multiple submitters, no conflicts
c.1012T>C (p.Cys338Arg)	Likely pathogenic	criteria provided, single submitter
c.1012T>A (p.Cys338Ser)	Pathogenic/Likely pathogenic	criteria provided, multiple submitters, no conflicts
c.1013G>T (p.Cys338Phe)	Likely pathogenic	criteria provided, single submitter
c.1013G>A (p.Cys338Tyr)	Pathogenic/Likely pathogenic	criteria provided, multiple submitters, no conflicts
c.1016T>C (p.Leu339Pro)	Likely pathogenic	criteria provided, multiple submitters, no conflicts
c.1019G>A (p.Cys340Tyr)	Conflicting interpretations of pathogenicity	criteria provided, conflicting interpretations
c.1020C>G (p.Cys340Trp)	Likely pathogenic	criteria provided, single submitter
c.1022C>G (p.Pro341Arg)	Likely benign	criteria provided, single submitter
c.1024G>T (p.Asp342Tyr)	Conflicting interpretations of pathogenicity	criteria provided, conflicting interpretations
c.1024G>A (p.Asp342Asn)	Conflicting interpretations of pathogenicity	criteria provided, conflicting interpretations
c.1026C>G (p.Asp342Glu)	Conflicting interpretations of pathogenicity	criteria provided, conflicting interpretations
c.1027G>T (p.Gly343Cys)	Likely pathogenic	criteria provided, single submitter

c.1027G>A (p.Gly343Ser)	Conflicting interpretations of pathogenicity	criteria provided, conflicting interpretations
c.1028G>T (p.Gly343Val)	Pathogenic	no assertion criteria provided
c.1028G>A (p.Gly343Asp)	Pathogenic/Likely pathogenic	criteria provided, multiple submitters, no conflicts
c.1036C>G (p.Leu346Val)	Uncertain significance	criteria provided, single submitter
c.1049G>A (p.Arg350Gln)	Pathogenic	no assertion criteria provided
c.1049G>C (p.Arg350Pro)	Conflicting interpretations of pathogenicity	criteria provided, conflicting interpretations
c.1054T>C (p.Cys352Arg)	Likely pathogenic	criteria provided, multiple submitters, no conflicts
c.1054T>A (p.Cys352Ser)	Likely pathogenic	criteria provided, multiple submitters, no conflicts
c.1055G>T (p.Cys352Phe)	Likely pathogenic	criteria provided, single submitter
c.1055G>A (p.Cys352Tyr)	Pathogenic/Likely pathogenic	criteria provided, multiple submitters, no conflicts
c.1056C>G (p.Cys352Trp)	Pathogenic/Likely pathogenic	criteria provided, multiple submitters, no conflicts
c.1057G>A (p.Glu353Lys)	Uncertain significance	criteria provided, multiple submitters, no conflicts

Table S2: LDLR variants within the EGF-B domain annotated at ClinVar (10/12/2019).

EGF-B	Clinical significance	Review status
c.1061A>T (p.Asp354Val)	Pathogenic/Likely pathogenic	criteria provided, multiple submitters, no conflicts
c.1061A>G (p.Asp354Gly)	Likely pathogenic	criteria provided, multiple submitters, no conflicts
c.1061A>C (p.Asp354Ala)	Likely pathogenic	criteria provided, multiple submitters, no conflicts
c.1063A>G (p.Ile355Val)	Likely benign	criteria provided, single submitter
c.1065C>G (p.Ile355Met)	Uncertain significance	criteria provided, single submitter
c.1066G>C (p.Asp356His)	Conflicting interpretations of pathogenicity	criteria provided, conflicting interpretations
c.1066G>A (p.Asp356Asn)	Conflicting interpretations of pathogenicity	criteria provided, conflicting interpretations
c.1066G>T (p.Asp356Tyr)	Conflicting interpretations of pathogenicity	criteria provided, conflicting interpretations
c.1067A>T (p.Asp356Val)	Pathogenic	no assertion criteria provided
c.1067A>C (p.Asp356Ala)	Likely pathogenic	criteria provided, multiple submitters, no conflicts
c.1069G>C (p.Glu357Gln)	Uncertain significance	criteria provided, single submitter
c.1069G>A (p.Glu357Lys)	Conflicting interpretations of pathogenicity	criteria provided, conflicting interpretations
c.1070A>G (p.Glu357Gly)	Likely pathogenic	criteria provided, single submitter
c.1072T>C (p.Cys358Arg)	Likely pathogenic	criteria provided, multiple submitters, no conflicts
c.1073G>A (p.Cys358Tyr)	Likely pathogenic	criteria provided, multiple submitters, no conflicts
c.1078G>C (p.Asp360His)	Uncertain significance	criteria provided, multiple submitters, no conflicts
c.1080T>G (p.Asp360Glu)	Uncertain significance	criteria provided, single submitter
**c.1081C>T (p.Pro361Ser)**	Pathogenic	criteria provided, single submitter
c.1085A>C (p.Asp362Ala)	Conflicting interpretations of pathogenicity	criteria provided, conflicting interpretations
c.1088C>A (p.Thr363Asn)	Conflicting interpretations of pathogenicity	criteria provided, conflicting interpretations
c.1090T>C (p.Cys364Arg)	Conflicting interpretations of pathogenicity	criteria provided, conflicting interpretations
c.1091G>A (p.Cys364Tyr)	Likely pathogenic	criteria provided, multiple submitters, no conflicts
c.1091G>T (p.Cys364Phe)	Likely pathogenic	criteria provided, single submitter
c.1091G>C (p.Cys364Ser)	Likely pathogenic	criteria provided, multiple submitters, no conflicts
c.1097A>G (p.Gln366Arg)	Likely pathogenic	criteria provided, single submitter
c.1097A>C (p.Gln366Pro)	Likely pathogenic	criteria provided, single submitter
c.1102T>C (p.Cys368Arg)	Pathogenic/Likely pathogenic	criteria provided, multiple submitters, no conflicts
c.1102T>G (p.Cys368Gly)	Likely pathogenic	criteria provided, single submitter
c.1103G>C (p.Cys368Ser)	Likely pathogenic	criteria provided, multiple submitters, no conflicts
c.1103G>A (p.Cys368Tyr)	Conflicting interpretations of pathogenicity	criteria provided, conflicting interpretations
c.1105G>A (p.Val369Met)	not provided	no assertion provided
c.1109A>C (p.Asn370Thr)	Pathogenic/Likely pathogenic	criteria provided, multiple submitters, no conflicts
c.1110C>G (p.Asn370Lys)	Likely pathogenic	criteria provided, single submitter
c.1112T>C (p.Leu371Pro)	Uncertain significance	criteria provided, single submitter
c.1117G>T (p.Gly373Cys)	Likely pathogenic	criteria provided, multiple submitters, no conflicts
c.1118G>C (p.Gly373Ala)	Uncertain significance	criteria provided, single submitter
c.1118G>T (p.Gly373Val)	Likely pathogenic	criteria provided, single submitter
c.1118G>A (p.Gly373Asp)	Pathogenic/Likely pathogenic	criteria provided, multiple submitters, no conflicts
c.1124A>G (p.Tyr375Cys)	Pathogenic/Likely pathogenic	criteria provided, multiple submitters, no conflicts
c.1124A>C (p.Tyr375Ser)	Likely pathogenic	criteria provided, single submitter
c.1129T>G (p.Cys377Gly)	Likely pathogenic	criteria provided, single submitter
c.1130G>T (p.Cys377Phe)	Likely pathogenic	criteria provided, single submitter
c.1130G>C (p.Cys377Ser)	Likely pathogenic	criteria provided, single submitter

c.1130G>A (p.Cys377Tyr)	Likely pathogenic	criteria provided, multiple submitters, no conflicts
c.1133A>C (p.Gln378Pro)	Conflicting interpretations of pathogenicity	criteria provided, conflicting interpretations
c.1135T>G (p.Cys379Gly)	Likely pathogenic	criteria provided, single submitter
c.1135T>C (p.Cys379Arg)	Pathogenic/Likely pathogenic	criteria provided, multiple submitters, no conflicts
c.1136G>A (p.Cys379Tyr)	Pathogenic/Likely pathogenic	criteria provided, multiple submitters, no conflicts
c.1145G>T (p.Gly382Val)	Likely pathogenic	criteria provided, single submitter
c.1151A>C (p.Gln384Pro)	Likely pathogenic	criteria provided, multiple submitters, no conflicts
c.1153C>G (p.Leu385Val)	Conflicting interpretations of pathogenicity	criteria provided, conflicting interpretations
c.1154T>C (p.Leu385Pro)	Pathogenic	no assertion criteria provided
c.1154T>G (p.Leu385Arg)	Likely pathogenic	criteria provided, single submitter
c.1156G>T (p.Asp386Tyr)	Uncertain significance	criteria provided, single submitter
c.1158C>G (p.Asp386Glu)	Conflicting interpretations of pathogenicity	criteria provided, conflicting interpretations
c.1166C>T (p.Thr389Met)	Conflicting interpretations of pathogenicity	criteria provided, conflicting interpretations
c.1171G>A (p.Ala391Thr)	Benign/Likely benign	criteria provided, multiple submitters, no conflicts
c.1174T>C (p.Cys392Arg)	Pathogenic/Likely pathogenic	criteria provided, multiple submitters, no conflicts
c.1175G>A (p.Cys392Tyr)	Conflicting interpretations of pathogenicity	criteria provided, conflicting interpretations
c.1177A>C (p.Lys393Gln)	Likely pathogenic	criteria provided, single submitter
c.1179G>C (p.Lys393Asn)	Likely pathogenic	criteria provided, single submitter
c.1186G>A (p.Gly396Ser)	Conflicting interpretations of pathogenicity	criteria provided, conflicting interpretations
c.1187G>A (p.Gly396Asp)	Pathogenic	no assertion criteria provided
c.1189T>A (p.Ser397Thr)	Likely benign	criteria provided, single submitter

Table S3: LDLR variants within the YWTD-1 domain annotated at ClinVar (10/12/2019).

YWTD-1	Clinical significance	Review status
c.1195G>T (p.Ala399Ser)	Pathogenic	no assertion criteria provided
c.1195G>A (p.Ala399Thr)	Likely pathogenic	criteria provided, single submitter
c.1196C>A (p.Ala399Asp)	Likely pathogenic	criteria provided, single submitter
c.1201C>G (p.Leu401Val)	Conflicting interpretations of pathogenicity	criteria provided, conflicting interpretations
c.1202T>A (p.Leu401His)	Likely pathogenic	criteria provided, single submitter
c.1205T>C (p.Phe402Ser)	Likely pathogenic	criteria provided, single submitter
c.1207T>C (p.Phe403Leu)	Likely pathogenic	criteria provided, single submitter
c.1209C>A (p.Phe403Leu)	Uncertain significance	criteria provided, single submitter
c.1210A>T (p.Thr404Ser)	Likely pathogenic	criteria provided, single submitter
c.1210A>C (p.Thr404Pro)	Conflicting interpretations of pathogenicity	criteria provided, conflicting interpretations
c.1211C>T (p.Thr404Ile)	Pathogenic/Likely pathogenic	criteria provided, multiple submitters, no conflicts
c.1214A>C (p.Asn405Thr)	Uncertain significance	criteria provided, single submitter
c.1215C>G (p.Asn405Lys)	Pathogenic/Likely pathogenic	criteria provided, multiple submitters, no conflicts
c.1216C>T (p.Arg406Trp)	Pathogenic/Likely pathogenic	criteria provided, multiple submitters, no conflicts
c.1217G>A (p.Arg406Gln)	Conflicting interpretations of pathogenicity	criteria provided, conflicting interpretations
c.1217G>C (p.Arg406Pro)	Likely pathogenic	criteria provided, multiple submitters, no conflicts
c.1222G>C (p.Glu408Gln)	Likely pathogenic	criteria provided, single submitter
c.1222G>A (p.Glu408Lys)	Pathogenic/Likely pathogenic	criteria provided, multiple submitters, no conflicts
c.1223A>T (p.Glu408Val)	Likely pathogenic	criteria provided, multiple submitters, no conflicts
c.1223A>C (p.Glu408Ala)	Likely pathogenic	criteria provided, multiple submitters, no conflicts
c.1224G>C (p.Glu408Asp)	Uncertain significance	criteria provided, single submitter
c.1230G>T (p.Arg410Ser)	Likely pathogenic	criteria provided, single submitter
c.1230G>C (p.Arg410Ser)	Conflicting interpretations of pathogenicity	criteria provided, conflicting interpretations
c.1231A>G (p.Lys411Glu)	Likely pathogenic	criteria provided, multiple submitters, no conflicts
c.1234A>C (p.Met412Leu)	Pathogenic	no assertion criteria provided
c.1235T>C (p.Met412Thr)	Pathogenic/Likely pathogenic	criteria provided, multiple submitters, no conflicts
c.1236G>A (p.Met412Ile)	Likely benign	criteria provided, single submitter
c.1238C>T (p.Thr413Met)	Conflicting interpretations of pathogenicity	criteria provided, conflicting interpretations
c.1241T>G (p.Leu414Arg)	Likely pathogenic	criteria provided, multiple submitters, no conflicts
c.1243G>C (p.Asp415His)	Pathogenic/Likely pathogenic	criteria provided, multiple submitters, no conflicts
c.1244A>T (p.Asp415Val)	Uncertain significance	criteria provided, single submitter
c.1244A>G (p.Asp415Gly)	Likely benign	criteria provided, single submitter
c.1246C>T (p.Arg416Trp)	Pathogenic/Likely pathogenic	criteria provided, multiple submitters, no conflicts
c.1247G>T (p.Arg416Leu)	Likely pathogenic	criteria provided, single submitter
c.1247G>C (p.Arg416Pro)	Likely pathogenic	criteria provided, multiple submitters, no conflicts
c.1247G>A (p.Arg416Gln)	Pathogenic/Likely pathogenic	criteria provided, multiple submitters, no conflicts
c.1252G>A (p.Glu418Lys)	Likely pathogenic	criteria provided, multiple submitters, no conflicts
c.1255T>G (p.Tyr419Asp)	Likely pathogenic	criteria provided, multiple submitters, no conflicts
c.1255T>C (p.Tyr419His)	Likely pathogenic	criteria provided, single submitter
c.1256A>G (p.Tyr419Cys)	Likely pathogenic	criteria provided, multiple submitters, no conflicts
c.1261A>G (p.Ser421Gly)	Likely benign	no assertion criteria provided
c.1265T>C (p.Leu422Pro)	Likely pathogenic	criteria provided, single submitter

c.1268T>C (p.Ile423Thr)	Pathogenic/Likely pathogenic	criteria provided, multiple submitters, no conflicts
c.1269C>G (p.Ile423Met)	Likely pathogenic	criteria provided, single submitter
c.1273A>G (p.Asn425Asp)	Uncertain significance	criteria provided, single submitter
c.1274A>T (p.Asn425Ile)	Likely pathogenic	criteria provided, single submitter
c.1277T>C (p.Leu426Pro)	Conflicting interpretations of pathogenicity	criteria provided, conflicting interpretations
c.1283A>G (p.Asn428Ser)	Uncertain significance	criteria provided, single submitter
c.1284C>G (p.Asn428Lys)	Uncertain significance	criteria provided, single submitter
c.1285G>T (p.Val429Leu)	Likely pathogenic	criteria provided, multiple submitters, no conflicts
c.1285G>C (p.Val429Leu)	Pathogenic/Likely pathogenic	criteria provided, multiple submitters, no conflicts
c.1285G>A (p.Val429Met)	Pathogenic/Likely pathogenic	criteria provided, multiple submitters, no conflicts
c.1288G>C (p.Val430Leu)	Conflicting interpretations of pathogenicity	criteria provided, conflicting interpretations
c.1291G>C (p.Ala431Pro)	Likely pathogenic	criteria provided, single submitter
c.1291G>A (p.Ala431Thr)	Pathogenic/Likely pathogenic	criteria provided, multiple submitters, no conflicts
c.1294C>G (p.Leu432Val)	Conflicting interpretations of pathogenicity	criteria provided, conflicting interpretations
c.1295T>C (p.Leu432Pro)	Likely pathogenic	criteria provided, single submitter
c.1297G>T (p.Asp433Tyr)	Likely pathogenic	criteria provided, single submitter
c.1297G>C (p.Asp433His)	Pathogenic/Likely pathogenic	criteria provided, multiple submitters, no conflicts
c.1300A>C (p.Thr434Pro)	Uncertain significance	criteria provided, single submitter
c.1301C>T (p.Thr434Met)	Conflicting interpretations of pathogenicity	criteria provided, conflicting interpretations
c.1301C>G (p.Thr434Arg)	Conflicting interpretations of pathogenicity	criteria provided, conflicting interpretations
c.1301C>A (p.Thr434Lys)	Conflicting interpretations of pathogenicity	criteria provided, conflicting interpretations
c.1307T>C (p.Val436Ala)	Conflicting interpretations of pathogenicity	criteria provided, conflicting interpretations

Table S4: LDLR variants within the YWTD-2 annotated at ClinVar (10/12/2019).

YWTD-2	Clinical significance	Review status
c.1315A>T (p.Asn439Tyr)	Likely pathogenic	criteria provided, multiple submitters, no conflicts
c.1317T>G (p.Asn439Lys)	Uncertain significance	criteria provided, single submitter
c.1318A>G (p.Arg440Gly)	Uncertain significance	criteria provided, single submitter
c.1322T>C (p.Ile441Thr)	Pathogenic/Likely pathogenic	criteria provided, multiple submitters, no conflicts
c.1322T>A (p.Ile441Asn)	Likely pathogenic	criteria provided, single submitter
c.1323C>G (p.Ile441Met)	Pathogenic/Likely pathogenic	criteria provided, multiple submitters, no conflicts
c.1324T>C (p.Tyr442His)	Likely pathogenic	criteria provided, single submitter
c.1324T>A (p.Tyr442Asn)	Likely pathogenic	criteria provided, single submitter
c.1325A>G (p.Tyr442Cys)	Likely pathogenic	criteria provided, multiple submitters, no conflicts
c.1328G>C (p.Trp443Ser)	Conflicting interpretations of pathogenicity	criteria provided, conflicting interpretations
c.1329G>T (p.Trp443Cys)	Likely pathogenic	criteria provided, multiple submitters, no conflicts
c.1329G>C (p.Trp443Cys)	Pathogenic/Likely pathogenic	criteria provided, multiple submitters, no conflicts
c.1330T>C (p.Ser444Pro)	Likely pathogenic	criteria provided, multiple submitters, no conflicts
c.1335C>G (p.Asp445Glu)	Uncertain significance	criteria provided, single submitter
c.1335C>A (p.Asp445Glu)	Likely pathogenic	criteria provided, single submitter
c.1336C>A (p.Leu446Met)	Uncertain significance	criteria provided, single submitter
c.1336C>G (p.Leu446Val)	not provided	no assertion provided
c.1339T>C (p.Ser447Pro)	Likely pathogenic	criteria provided, single submitter
c.1340C>G (p.Ser447Cys)	Likely pathogenic	criteria provided, single submitter
c.1342C>A (p.Gln448Lys)	Pathogenic	no assertion criteria provided
c.1351A>T (p.Ile451Phe)	Likely pathogenic	criteria provided, single submitter
c.1352T>C (p.Ile451Thr)	Likely pathogenic	criteria provided, multiple submitters, no conflicts
c.1361C>A (p.Thr454Asn)	Likely pathogenic	criteria provided, multiple submitters, no conflicts
c.1367T>C (p.Leu456Pro)	Uncertain significance	criteria provided, single submitter
c.1367T>A (p.Leu456His)	Likely pathogenic	criteria provided, single submitter
c.1376C>G (p.Ala459Gly)	Likely benign	criteria provided, single submitter
c.1381G>T (p.Gly461Cys)	Uncertain significance	criteria provided, single submitter
c.1381G>A (p.Gly461Ser)	Likely pathogenic	criteria provided, single submitter
c.1384G>A (p.Val462Ile)	Likely benign	criteria provided, single submitter
c.1393T>A (p.Tyr465Asn)	Conflicting interpretations of pathogenicity	criteria provided, conflicting interpretations
c.1394A>G (p.Tyr465Cys)	Conflicting interpretations of pathogenicity	criteria provided, conflicting interpretations
c.1400C>T (p.Thr467Ile)	Likely benign	criteria provided, single submitter
c.1402G>A (p.Val468Ile)	Likely benign	criteria provided, multiple submitters, no conflicts
c.1403T>A (p.Val468Asp)	Conflicting interpretations of pathogenicity	criteria provided, conflicting interpretations
c.1408A>T (p.Ser470Cys)	Uncertain significance	criteria provided, single submitter
c.1408A>G (p.Ser470Gly)	Likely pathogenic	criteria provided, single submitter
c.1411A>G (p.Arg471Gly)	Likely benign	criteria provided, single submitter
c.1412G>A (p.Arg471Lys)	Likely pathogenic	criteria provided, single submitter
c.1414G>A (p.Asp472Asn)	Uncertain significance	criteria provided, single submitter
c.1414G>T (p.Asp472Tyr)	Conflicting interpretations of pathogenicity	criteria provided, conflicting interpretations
c.1417A>G (p.Ile473Val)	Benign/Likely benign	criteria provided, multiple submitters, no conflicts
c.1418T>G (p.Ile473Ser)	Uncertain significance	criteria provided, single submitter
c.1418T>A (p.Ile473Asn)	Conflicting interpretations of pathogenicity	criteria provided, conflicting interpretations

c.1424C>T (p.Ala475Val)	Likely pathogenic	criteria provided, multiple submitters, no conflicts
c.1426C>T (p.Pro476Ser)	Likely pathogenic	criteria provided, single submitter
c.1427C>T (p.Pro476Leu)	Pathogenic	criteria provided, single submitter
c.1429G>A (p.Asp477Asn)	Uncertain significance	criteria provided, multiple submitters, no conflicts
c.1432G>T (p.Gly478Trp)	Uncertain significance	criteria provided, single submitter
c.1432G>A (p.Gly478Arg)	Conflicting interpretations of pathogenicity	criteria provided, conflicting interpretations
c.1433G>A (p.Gly478Glu)	Pathogenic	no assertion criteria provided
c.1435C>G (p.Leu479Val)	Pathogenic	no assertion criteria provided
c.1436T>C (p.Leu479Pro)	Likely pathogenic	criteria provided, multiple submitters, no conflicts
c.1438G>A (p.Ala480Thr)	Pathogenic	no assertion criteria provided
c.1438G>C (p.Ala480Pro)	Likely pathogenic	criteria provided, single submitter
**c.1439C>T (p.Ala480Val)**	Pathogenic	criteria provided, single submitter
c.1441G>A (p.Val481Met)	Likely pathogenic	criteria provided, single submitter
c.1444G>T (p.Asp482Tyr)	Likely pathogenic	criteria provided, single submitter
c.1444G>C (p.Asp482His)	Likely pathogenic	criteria provided, single submitter
c.1444G>A (p.Asp482Asn)	Pathogenic/Likely pathogenic	criteria provided, multiple submitters, no conflicts
c.1445A>G (p.Asp482Gly)	Likely pathogenic	criteria provided, single submitter
c.1447T>C (p.Trp483Arg)	Likely pathogenic	criteria provided, multiple submitters, no conflicts
c.1448G>T (p.Trp483Leu)	Pathogenic	no assertion criteria provided
c.1449G>C (p.Trp483Cys)	Uncertain significance	criteria provided, single submitter
c.1449G>T (p.Trp483Cys)	Uncertain significance	criteria provided, single submitter
c.1454A>G (p.His485Arg)	Likely pathogenic	criteria provided, single submitter
c.1455C>G (p.His485Gln)	Conflicting interpretations of pathogenicity	criteria provided, conflicting interpretations

Table S5: LDLR variants within the YWTD-3 annotated at ClinVar (10/12/2019).

YWTD-3	Clinical significance	Review status
c.1456A>G (p.Ser486Gly)	Likely benign	criteria provided, single submitter
c.1457G>A (p.Ser486Asn)	Likely benign	criteria provided, single submitter
c.1460A>G (p.Asn487Ser)	Conflicting interpretations of pathogenicity	criteria provided, conflicting interpretations
c.1463T>G (p.Ile488Ser)	Likely pathogenic	criteria provided, single submitter
c.1463T>C (p.Ile488Thr)	Conflicting interpretations of pathogenicity	criteria provided, conflicting interpretations
c.1463T>A (p.Ile488Asn)	Pathogenic/Likely pathogenic	criteria provided, multiple submitters, no conflicts
c.1466A>G (p.Tyr489Cys)	Likely pathogenic	criteria provided, single submitter
c.1468T>G (p.Trp490Gly)	Likely pathogenic	criteria provided, single submitter
c.1468T>C (p.Trp490Arg)	Conflicting interpretations of pathogenicity	criteria provided, conflicting interpretations
c.1469G>T (p.Trp490Leu)	Pathogenic	no assertion criteria provided
c.1472C>A (p.Thr491Asn)	Likely pathogenic	criteria provided, single submitter
c.1474G>C (p.Asp492His)	Conflicting interpretations of pathogenicity	criteria provided, conflicting interpretations
c.1474G>A (p.Asp492Asn)	Conflicting interpretations of pathogenicity	criteria provided, conflicting interpretations
c.1475A>G (p.Asp492Gly)	Likely pathogenic	criteria provided, multiple submitters, no conflicts
c.1487G>T (p.Gly496Val)	Likely pathogenic	criteria provided, multiple submitters, no conflicts
c.1489A>C (p.Thr497Pro)	Likely pathogenic	criteria provided, multiple submitters, no conflicts
c.1492G>T (p.Val498Phe)	Likely benign	criteria provided, single submitter
c.1495T>C (p.Ser499Pro)	Conflicting interpretations of pathogenicity	criteria provided, conflicting interpretations
c.1499T>C (p.Val500Ala)	Uncertain significance	criteria provided, single submitter
c.1502C>T (p.Ala501Val)	Pathogenic/Likely pathogenic	criteria provided, multiple submitters, no conflicts
c.1502C>A (p.Ala501Glu)	Likely pathogenic	criteria provided, single submitter
c.1504G>T (p.Asp502Tyr)	Uncertain significance	criteria provided, single submitter
c.1510A>G (p.Lys504Glu)	Conflicting interpretations of pathogenicity	criteria provided, conflicting interpretations
c.1514G>A (p.Gly505Asp)	Likely pathogenic	criteria provided, single submitter
c.1516G>A (p.Val506Met)	Conflicting interpretations of pathogenicity	criteria provided, conflicting interpretations
c.1519A>G (p.Lys507Glu)	Uncertain significance	criteria provided, single submitter
c.1520A>C (p.Lys507Thr)	Likely pathogenic	criteria provided, single submitter
c.1521G>C (p.Lys507Asn)	Pathogenic	no assertion criteria provided
c.1525A>G (p.Lys509Glu)	Likely pathogenic	criteria provided, single submitter
c.1529C>T (p.Thr510Met)	Likely pathogenic	criteria provided, multiple submitters, no conflicts
c.1532T>C (p.Leu511Ser)	Likely pathogenic	criteria provided, single submitter
c.1533A>T (p.Leu511Phe)	Uncertain significance	criteria provided, single submitter
c.1538G>A (p.Arg513Lys)	Likely benign	criteria provided, multiple submitters, no conflicts
c.1546G>A (p.Gly516Ser)	Conflicting interpretations of pathogenicity	criteria provided, conflicting interpretations
c.1547G>A (p.Gly516Asp)	not provided	no assertion provided
c.1549T>C (p.Ser517Pro)	Conflicting interpretations of pathogenicity	criteria provided, conflicting interpretations
c.1552A>G (p.Lys518Glu)	Conflicting interpretations of pathogenicity	criteria provided, conflicting interpretations
c.1553A>G (p.Lys518Arg)	Likely benign	criteria provided, single submitter
c.1555C>T (p.Pro519Ser)	Likely pathogenic	criteria provided, multiple submitters, no conflicts
c.1555C>A (p.Pro519Thr)	Pathogenic	no assertion criteria provided
c.1558A>G (p.Arg520Gly)	Pathogenic/Likely pathogenic	criteria provided, multiple submitters, no conflicts
c.1561G>A (p.Ala521Thr)	Uncertain significance	criteria provided, multiple submitters, no conflicts
c.1562C>T (p.Ala521Val)	Uncertain significance	criteria provided, single submitter

c.1564A>C (p.Ile522Leu)	Uncertain significance	criteria provided, single submitter
c.1565T>G (p.Ile522Ser)	Uncertain significance	criteria provided, single submitter
c.1567G>T (p.Val523Leu)	Conflicting interpretations of pathogenicity	criteria provided, conflicting interpretations
c.1567G>A (p.Val523Met)	Pathogenic/Likely pathogenic	criteria provided, multiple submitters, no conflicts
c.1570G>A (p.Val524Met)	Conflicting interpretations of pathogenicity	criteria provided, conflicting interpretations
c.1571T>G (p.Val524Gly)	Likely pathogenic	criteria provided, multiple submitters, no conflicts
c.1574A>C (p.Asp525Ala)	Uncertain significance	criteria provided, single submitter
c.1574A>T (p.Asp525Val)	Likely pathogenic	criteria provided, single submitter
c.1576C>A (p.Pro526Thr)	Likely pathogenic	criteria provided, single submitter
c.1576C>T (p.Pro526Ser)	Conflicting interpretations of pathogenicity	criteria provided, conflicting interpretations
c.1577C>A (p.Pro526His)	Uncertain significance	criteria provided, single submitter
c.1577C>T (p.Pro526Leu)	Uncertain significance	criteria provided, single submitter
c.1577C>G (p.Pro526Arg)	Likely pathogenic	criteria provided, single submitter
c.1580T>C (p.Val527Ala)	not provided	no assertion provided

Table S6: LDLR variants within the YWTD-4 annotated at ClinVar (10/12/2019).

YWTD-4	Clinical significance	Review status
c.1585G>C (p.Gly529Arg)	Conflicting interpretations of pathogenicity	criteria provided, conflicting interpretations
c.1586G>A (p.Gly529Asp)	Likely pathogenic	criteria provided, multiple submitters, no conflicts
c.1588T>G (p.Phe530Val)	Uncertain significance	criteria provided, single submitter
c.1592T>G (p.Met531Arg)	Likely pathogenic	criteria provided, single submitter
c.1594T>A (p.Tyr532Asn)	Pathogenic	no assertion criteria provided
c.1595A>G (p.Tyr532Cys)	Uncertain significance	criteria provided, single submitter
c.1597T>C (p.Trp533Arg)	Likely pathogenic	criteria provided, multiple submitters, no conflicts
c.1601C>A (p.Thr534Asn)	Likely pathogenic	criteria provided, single submitter
c.1603G>A (p.Asp535Asn)	Pathogenic	no assertion criteria provided
c.1603G>T (p.Asp535Tyr)	Likely pathogenic	criteria provided, single submitter
c.1606T>G (p.Trp536Gly)	Likely pathogenic	criteria provided, multiple submitters, no conflicts
c.1612A>T (p.Thr538Ser)	Likely benign	criteria provided, single submitter
c.1618G>T (p.Ala540Ser)	Uncertain significance	criteria provided, single submitter
c.1618G>A (p.Ala540Thr)	Pathogenic/Likely pathogenic	criteria provided, multiple submitters, no conflicts
c.1625T>G (p.Ile542Ser)	Pathogenic/Likely pathogenic	criteria provided, multiple submitters, no conflicts
c.1633G>T (p.Gly545Trp)	Likely pathogenic	criteria provided, multiple submitters, no conflicts
c.1633G>C (p.Gly545Arg)	Likely pathogenic	criteria provided, single submitter
c.1633G>A (p.Gly545Arg)	Pathogenic/Likely pathogenic	criteria provided, multiple submitters, no conflicts
c.1634G>A (p.Gly545Glu)	Conflicting interpretations of pathogenicity	criteria provided, conflicting interpretations
c.1636G>C (p.Gly546Arg)	Likely pathogenic	criteria provided, single submitter
c.1637G>T (p.Gly546Val)	Likely pathogenic	criteria provided, multiple submitters, no conflicts
c.1637G>A (p.Gly546Asp)	Pathogenic/Likely pathogenic	criteria provided, multiple submitters, no conflicts
c.1640T>C (p.Leu547Pro)	Conflicting interpretations of pathogenicity	criteria provided, conflicting interpretations
c.1644T>G (p.Asn548Lys)	Likely pathogenic	criteria provided, multiple submitters, no conflicts
c.1644T>A (p.Asn548Lys)	Likely pathogenic	criteria provided, single submitter
c.1646G>T (p.Gly549Val)	Likely pathogenic	criteria provided, single submitter
c.1646G>A (p.Gly549Asp)	Pathogenic/Likely pathogenic	criteria provided, multiple submitters, no conflicts
c.1658A>G (p.Tyr553Cys)	Uncertain significance	criteria provided, single submitter
c.1661C>T (p.Ser554Leu)	Conflicting interpretations of pathogenicity	criteria provided, conflicting interpretations
c.1664T>C (p.Leu555Pro)	Likely pathogenic	criteria provided, multiple submitters, no conflicts
c.1678A>T (p.Ile560Phe)	Pathogenic	criteria provided, single submitter
c.1687C>T (p.Pro563Ser)	Likely pathogenic	criteria provided, multiple submitters, no conflicts
c.1688C>A (p.Pro563His)	Likely pathogenic	criteria provided, single submitter
c.1690A>G (p.Asn564Asp)	Pathogenic/Likely pathogenic	criteria provided, multiple submitters, no conflicts
c.1690A>C (p.Asn564His)	Conflicting interpretations of pathogenicity	criteria provided, conflicting interpretations
c.1691A>G (p.Asn564Ser)	Likely pathogenic	criteria provided, multiple submitters, no conflicts
c.1694G>A (p.Gly565Asp)	Likely pathogenic	criteria provided, single submitter
c.1694G>C (p.Gly565Ala)	Pathogenic/Likely pathogenic	criteria provided, multiple submitters, no conflicts
c.1694G>T (p.Gly565Val)	Pathogenic/Likely pathogenic	criteria provided, multiple submitters, no conflicts
c.1700C>A (p.Thr567Asn)	Uncertain significance	criteria provided, single submitter
c.1702C>G (p.Leu568Val)	Pathogenic/Likely pathogenic	criteria provided, multiple submitters, no conflicts
c.1703T>C (p.Leu568Pro)	Pathogenic/Likely pathogenic	criteria provided, multiple submitters, no conflicts

c.1705G>A (p.Asp569Asn)	Likely pathogenic	criteria provided, single submitter
c.1705G>T (p.Asp569Tyr)	Likely pathogenic	criteria provided, multiple submitters, no conflicts
c.1715G>A (p.Ser572Asn)	Likely benign	criteria provided, multiple submitters, no conflicts

Table S7: LDLR variants within the YWTD-5 annotated at ClinVar (10/12/2019).

YWTD-5	Clinical significance	Review status
c.1720C>T (p.Arg574Cys)	Conflicting interpretations of pathogenicity	criteria provided, conflicting interpretations
c.1721G>A (p.Arg574His)	Conflicting interpretations of pathogenicity	criteria provided, conflicting interpretations
c.1721G>T (p.Arg574Leu)	Conflicting interpretations of pathogenicity	criteria provided, conflicting interpretations
c.1723C>T (p.Leu575Phe)	Likely pathogenic	criteria provided, multiple submitters, no conflicts
c.1727A>G (p.Tyr576Cys)	Likely pathogenic	criteria provided, single submitter
c.1727A>C (p.Tyr576Ser)	Likely pathogenic	criteria provided, single submitter
c.1729T>C (p.Trp577Arg)	Pathogenic/Likely pathogenic	criteria provided, multiple submitters, no conflicts
c.1729T>G (p.Trp577Gly)	Pathogenic/Likely pathogenic	criteria provided, multiple submitters, no conflicts
c.1730G>C (p.Trp577Ser)	Pathogenic/Likely pathogenic	criteria provided, multiple submitters, no conflicts
c.1731G>C (p.Trp577Cys)	Pathogenic	criteria provided, single submitter
c.1731G>T (p.Trp577Cys)	Conflicting interpretations of pathogenicity	criteria provided, conflicting interpretations
c.1733T>C (p.Val578Ala)	Pathogenic	criteria provided, single submitter
c.1735G>A (p.Asp579Asn)	Pathogenic/Likely pathogenic	criteria provided, multiple submitters, no conflicts
c.1735G>T (p.Asp579Tyr)	Likely pathogenic	criteria provided, multiple submitters, no conflicts
c.1736A>G (p.Asp579Gly)	Likely pathogenic	criteria provided, multiple submitters, no conflicts
c.1736A>C (p.Asp579Ala)	Likely pathogenic	criteria provided, single submitter
c.1737C>G (p.Asp579Glu)	Likely pathogenic	criteria provided, single submitter
c.1738T>C (p.Ser580Pro)	Likely pathogenic	criteria provided, single submitter
c.1739C>T (p.Ser580Phe)	Likely pathogenic	criteria provided, single submitter
c.1743A>T (p.Lys581Asn)	Pathogenic	no assertion criteria provided
c.1744C>G (p.Leu582Val)	Uncertain significance	criteria provided, single submitter
c.1744C>T (p.Leu582Phe)	Pathogenic/Likely pathogenic	criteria provided, multiple submitters, no conflicts
c.1745T>C (p.Leu582Pro)	Likely pathogenic	criteria provided, multiple submitters, no conflicts
c.1747C>T (p.His583Tyr)	Conflicting interpretations of pathogenicity	criteria provided, conflicting interpretations
c.1747C>G (p.His583Asp)	Likely pathogenic	criteria provided, single submitter
c.1748A>G (p.His583Arg)	Likely pathogenic	criteria provided, multiple submitters, no conflicts
c.1749C>G (p.His583Gln)	Likely pathogenic	criteria provided, single submitter
c.1749C>A (p.His583Gln)	Likely pathogenic	criteria provided, single submitter
c.1750T>C (p.Ser584Pro)	Conflicting interpretations of pathogenicity	criteria provided, conflicting interpretations
c.1754T>C (p.Ile585Thr)	Likely pathogenic	criteria provided, single submitter
c.1754T>A (p.Ile585Asn)	Likely pathogenic	criteria provided, single submitter
c.1761C>G (p.Ser587Arg)	Uncertain significance	criteria provided, multiple submitters, no conflicts
c.1765G>T (p.Asp589Tyr)	Uncertain significance	criteria provided, single submitter
c.1765G>A (p.Asp589Asn)	Uncertain significance	criteria provided, multiple submitters, no conflicts
c.1765G>C (p.Asp589His)	not provided	no assertion provided
c.1771A>G (p.Asn591Asp)	Uncertain significance	criteria provided, single submitter
c.1774G>A (p.Gly592Arg)	Pathogenic/Likely pathogenic	criteria provided, multiple submitters, no conflicts
c.1775G>A (p.Gly592Glu)	Pathogenic/Likely pathogenic	criteria provided, multiple submitters, no conflicts
c.1783C>T (p.Arg595Trp)	Conflicting interpretations of pathogenicity	criteria provided, conflicting interpretations
c.1784G>T (p.Arg595Leu)	Likely pathogenic	criteria provided, single submitter
c.1784G>A (p.Arg595Gln)	Conflicting interpretations of pathogenicity	criteria provided, conflicting interpretations
c.1792A>C (p.Ile598Leu)	Likely benign	criteria provided, single submitter
c.1793T>C (p.Ile598Thr)	Uncertain significance	criteria provided, single submitter

c.1793T>A (p.Ile598Asn)	Uncertain significance	criteria provided, single submitter
c.1796T>G (p.Leu599Trp)	Likely pathogenic	criteria provided, single submitter
c.1796T>C (p.Leu599Ser)	Conflicting interpretations of pathogenicity	criteria provided, conflicting interpretations
c.1800G>C (p.Glu600Asp)	Conflicting interpretations of pathogenicity	criteria provided, conflicting interpretations
c.1801G>T (p.Asp601Tyr)	Likely pathogenic	criteria provided, single submitter
c.1801G>C (p.Asp601His)	Conflicting interpretations of pathogenicity	criteria provided, conflicting interpretations
c.1802A>G (p.Asp601Gly)	Benign	no assertion criteria provided
c.1802A>T (p.Asp601Val)	Likely pathogenic	criteria provided, multiple submitters, no conflicts
c.1808A>G (p.Lys603Arg)	Benign	no assertion criteria provided
c.1809G>C (p.Lys603Asn)	Likely benign	criteria provided, single submitter
c.1814T>C (p.Leu605Pro)	Likely pathogenic	criteria provided, multiple submitters, no conflicts
c.1816G>A (p.Ala606Thr)	Conflicting interpretations of pathogenicity	criteria provided, conflicting interpretations
c.1816G>T (p.Ala606Ser)	Uncertain significance	criteria provided, multiple submitters, no conflicts
c.1817C>A (p.Ala606Asp)	Pathogenic	no assertion criteria provided
c.1819C>T (p.His607Tyr)	Uncertain significance	criteria provided, single submitter
c.1820A>G (p.His607Arg)	Likely pathogenic	criteria provided, single submitter
c.1822C>T (p.Pro608Ser)	Likely pathogenic	criteria provided, multiple submitters, no conflicts
c.1822C>A (p.Pro608Thr)	Likely pathogenic	criteria provided, single submitter
c.1823C>T (p.Pro608Leu)	Likely pathogenic	criteria provided, single submitter
c.1823C>G (p.Pro608Arg)	Likely pathogenic	criteria provided, multiple submitters, no conflicts
c.1825T>C (p.Phe609Leu)	Likely benign	criteria provided, single submitter
c.1829C>T (p.Ser610Phe)	Pathogenic/Likely pathogenic	criteria provided, multiple submitters, no conflicts
c.1829C>G (p.Ser610Cys)	Likely pathogenic	criteria provided, multiple submitters, no conflicts
c.1833G>T (p.Leu611Phe)	Likely pathogenic	criteria provided, single submitter
c.1833G>C (p.Leu611Phe)	Likely pathogenic	criteria provided, multiple submitters, no conflicts
c.1834G>T (p.Ala612Ser)	Likely benign	criteria provided, single submitter
c.1835C>T (p.Ala612Val)	Pathogenic	no assertion criteria provided
c.1837G>A (p.Val613Ile)	Conflicting interpretations of pathogenicity	criteria provided, conflicting interpretations
c.1840T>A (p.Phe614Ile)	Conflicting interpretations of pathogenicity	criteria provided, conflicting interpretations
c.1843G>A (p.Glu615Lys)	Conflicting interpretations of pathogenicity	criteria provided, conflicting interpretations
c.1844A>T (p.Glu615Val)	Likely pathogenic	criteria provided, multiple submitters, no conflicts

Table S8: LDLR variants within the YWTD-6 domain annotated at ClinVar (10/12/2019).

YWTD-6	Clinical significance	Review status
c.1853T>G (p.Val618Gly)	Pathogenic/Likely pathogenic	criteria provided, multiple submitters, no conflicts
c.1855T>C (p.Phe619Leu)	Conflicting interpretations of pathogenicity	criteria provided, conflicting interpretations
c.1856T>G (p.Phe619Cys)	Pathogenic/Likely pathogenic	criteria provided, multiple submitters, no conflicts
c.1856T>C (p.Phe619Ser)	Pathogenic/Likely pathogenic	criteria provided, multiple submitters, no conflicts
c.1858T>C (p.Trp620Arg)	Likely pathogenic	criteria provided, single submitter
c.1860G>C (p.Trp620Cys)	Likely pathogenic	criteria provided, multiple submitters, no conflicts
c.1860G>T (p.Trp620Cys)	Likely pathogenic	criteria provided, single submitter
c.1862C>G (p.Thr621Arg)	Pathogenic/Likely pathogenic	criteria provided, multiple submitters, no conflicts
c.1864G>A (p.Asp622Asn)	Pathogenic/Likely pathogenic	criteria provided, multiple submitters, no conflicts
c.1865A>G (p.Asp622Gly)	Conflicting interpretations of pathogenicity	criteria provided, conflicting interpretations
c.1865A>C (p.Asp622Ala)	Likely pathogenic	criteria provided, single submitter
c.1867A>G (p.Ile623Val)	Conflicting interpretations of pathogenicity	criteria provided, conflicting interpretations
c.1868T>C (p.Ile623Thr)	Uncertain significance	criteria provided, multiple submitters, no conflicts
c.1874A>C (p.Asn625Thr)	Conflicting interpretations of pathogenicity	criteria provided, conflicting interpretations
c.1876G>A (p.Glu626Lys)	Conflicting interpretations of pathogenicity	criteria provided, conflicting interpretations
c.1879G>A (p.Ala627Thr)	Likely pathogenic	criteria provided, multiple submitters, no conflicts
c.1880C>T (p.Ala627Val)	Likely pathogenic	criteria provided, multiple submitters, no conflicts
c.1880C>A (p.Ala627Asp)	Pathogenic/Likely pathogenic	criteria provided, multiple submitters, no conflicts
c.1883T>C (p.Ile628Thr)	Likely pathogenic	criteria provided, single submitter
c.1885T>G (p.Phe629Val)	Uncertain significance	criteria provided, single submitter
c.1886T>G (p.Phe629Cys)	Likely pathogenic	criteria provided, single submitter
c.1892C>A (p.Ala631Asp)	Likely pathogenic	criteria provided, single submitter
c.1895A>T (p.Asn632Ile)	Uncertain significance	criteria provided, single submitter
c.1897C>T (p.Arg633Cys)	Pathogenic/Likely pathogenic	criteria provided, multiple submitters, no conflicts
c.1898G>T (p.Arg633Leu)	Likely pathogenic	criteria provided, single submitter
c.1898G>A (p.Arg633His)	Conflicting interpretations of pathogenicity	criteria provided, conflicting interpretations
c.1900C>T (p.Leu634Phe)	Uncertain significance	criteria provided, multiple submitters, no conflicts
c.1904C>T (p.Thr635Ile)	Likely pathogenic	criteria provided, single submitter
c.1906G>A (p.Gly636Ser)	Likely pathogenic	criteria provided, single submitter
c.1907G>T (p.Gly636Val)	Likely pathogenic	criteria provided, single submitter
c.1907G>A (p.Gly636Asp)	Conflicting interpretations of pathogenicity	criteria provided, conflicting interpretations
c.1912G>A (p.Asp638Asn)	Uncertain significance	criteria provided, single submitter
c.1916T>G (p.Val639Gly)	Likely pathogenic	criteria provided, single submitter
c.1916T>A (p.Val639Asp)	Conflicting interpretations of pathogenicity	criteria provided, conflicting interpretations
c.1925T>C (p.Leu642Ser)	Likely pathogenic	criteria provided, single submitter
c.1927G>C (p.Ala643Pro)	Uncertain significance	criteria provided, single submitter
c.1928C>T (p.Ala643Val)	Conflicting interpretations of pathogenicity	criteria provided, conflicting interpretations
c.1936C>A (p.Leu646Ile)	Likely pathogenic	criteria provided, single submitter
c.1937T>A (p.Leu646Gln)	Pathogenic	no assertion criteria provided
c.1942T>G (p.Ser648Ala)	Likely pathogenic	criteria provided, single submitter
c.1942T>C (p.Ser648Pro)	Likely pathogenic	criteria provided, multiple submitters, no conflicts
**c.1943C>T (p.Ser648Phe)**	Pathogenic	no assertion criteria provided
c.1945C>T (p.Pro649Ser)	Likely pathogenic	criteria provided, single submitter

c.1946C>T (p.Pro649Leu)	Conflicting interpretations of pathogenicity	criteria provided, conflicting interpretations
c.1948G>A (p.Glu650Lys)	Uncertain significance	criteria provided, single submitter
c.1951G>T (p.Asp651Tyr)	Likely pathogenic	criteria provided, single submitter
c.1951G>A (p.Asp651Asn)	Conflicting interpretations of pathogenicity	criteria provided, conflicting interpretations
c.1952A>T (p.Asp651Val)	Likely pathogenic	criteria provided, single submitter
c.1954A>G (p.Met652Val)	Likely benign	criteria provided, single submitter
c.1955T>C (p.Met652Thr)	Conflicting interpretations of pathogenicity	criteria provided, conflicting interpretations
c.1957G>A (p.Val653Ile)	Uncertain significance	criteria provided, single submitter
c.1957G>T (p.Val653Phe)	Conflicting interpretations of pathogenicity	criteria provided, conflicting interpretations
c.1958T>G (p.Val653Gly)	Conflicting interpretations of pathogenicity	criteria provided, conflicting interpretations
c.1960C>T (p.Leu654Phe)	Conflicting interpretations of pathogenicity	criteria provided, conflicting interpretations
c.1965C>G (p.Phe655Leu)	Conflicting interpretations of pathogenicity	criteria provided, conflicting interpretations
c.1966C>A (p.His656Asn)	Conflicting interpretations of pathogenicity	criteria provided, conflicting interpretations
c.1968C>G (p.His656Gln)	Likely pathogenic	criteria provided, single submitter
c.1971C>A (p.Asn657Lys)	Uncertain significance	criteria provided, single submitter
c.1973T>C (p.Leu658Pro)	Likely pathogenic	criteria provided, multiple submitters, no conflicts
c.1975A>C (p.Thr659Pro)	Conflicting interpretations of pathogenicity	criteria provided, conflicting interpretations
c.1976C>A (p.Thr659Asn)	Likely benign	criteria provided, single submitter
c.1978C>A (p.Gln660Lys)	Likely pathogenic	criteria provided, single submitter
c.1979A>C (p.Gln660Pro)	Likely pathogenic	criteria provided, single submitter
c.1979A>G (p.Gln660Arg)	Likely pathogenic	criteria provided, single submitter
c.1981C>A (p.Pro661Thr)	Uncertain significance	criteria provided, single submitter
c.1982C>T (p.Pro661Leu)	Conflicting interpretations of pathogenicity	criteria provided, conflicting interpretations
c.1985G>A (p.Arg662Lys)	Uncertain significance	criteria provided, single submitter
c.1987G>A (p.Gly663Arg)	Uncertain significance	criteria provided, single submitter
c.1988G>C (p.Gly663Ala)	Pathogenic	criteria provided, single submitter

Table S9: LDLR variants within the EGF-C domain annotated at ClinVar (10/12/2019).

EGF-C	Clinical significance	Review status
c.1999T>C (p.Cys667Arg)	Pathogenic/Likely pathogenic	criteria provided, multiple submitters, no conflicts
c.2000G>A (p.Cys667Tyr)	Pathogenic/Likely pathogenic	criteria provided, multiple submitters, no conflicts
c.2001T>G (p.Cys667Trp)	Pathogenic/Likely pathogenic	criteria provided, multiple submitters, no conflicts
c.2023G>A (p.Gly675Ser)	Conflicting interpretations of pathogenicity	criteria provided, conflicting interpretations
c.2026G>T (p.Gly676Cys)	Pathogenic	no assertion criteria provided
c.2026G>C (p.Gly676Arg)	Likely pathogenic	criteria provided, single submitter
c.2026G>A (p.Gly676Ser)	Conflicting interpretations of pathogenicity	criteria provided, conflicting interpretations
c.2029T>C (p.Cys677Arg)	Pathogenic/Likely pathogenic	criteria provided, multiple submitters, no conflicts
c.2030G>T (p.Cys677Phe)	Likely pathogenic	criteria provided, single submitter
c.2039T>C (p.Leu680Pro)	Pathogenic	no assertion criteria provided
c.2041T>G (p.Cys681Gly)	Conflicting interpretations of pathogenicity	criteria provided, conflicting interpretations
c.2042G>A (p.Cys681Tyr)	Pathogenic/Likely pathogenic	criteria provided, multiple submitters, no conflicts
c.2044C>T (p.Leu682Phe)	Pathogenic	criteria provided, single submitter
c.2048C>T (p.Pro683Leu)	Uncertain significance	criteria provided, multiple submitters, no conflicts
c.2050G>A (p.Ala684Thr)	Likely pathogenic	criteria provided, multiple submitters, no conflicts
c.2054C>T (p.Pro685Leu)	Pathogenic/Likely pathogenic	criteria provided, multiple submitters, no conflicts
c.2056C>G (p.Gln686Glu)	Likely pathogenic	criteria provided, single submitter
c.2059A>T (p.Ile687Phe)	Uncertain significance	criteria provided, single submitter
c.2068C>G (p.His690Asp)	Uncertain significance	criteria provided, single submitter
c.2072C>T (p.Ser691Leu)	Uncertain significance	criteria provided, single submitter
c.2075C>G (p.Pro692Arg)	Uncertain significance	criteria provided, single submitter
c.2087G>A (p.Cys696Tyr)	Pathogenic/Likely pathogenic	criteria provided, multiple submitters, no conflicts
c.2089G>A (p.Ala697Thr)	Pathogenic	no assertion criteria provided
c.2092T>G (p.Cys698Gly)	Uncertain significance	criteria provided, single submitter
c.2093G>T (p.Cys698Phe)	Pathogenic/Likely pathogenic	criteria provided, multiple submitters, no conflicts
c.2093G>A (p.Cys698Tyr)	Likely pathogenic	criteria provided, multiple submitters, no conflicts
c.2094C>G (p.Cys698Trp)	Conflicting interpretations of pathogenicity	criteria provided, conflicting interpretations
c.2096C>T (p.Pro699Leu)	Conflicting interpretations of pathogenicity	criteria provided, conflicting interpretations
c.2098G>A (p.Asp700Asn)	Uncertain significance	criteria provided, multiple submitters, no conflicts
c.2099A>G (p.Asp700Gly)	Conflicting interpretations of pathogenicity	criteria provided, conflicting interpretations
c.2101G>A (p.Gly701Ser)	Conflicting interpretations of pathogenicity	criteria provided, conflicting interpretations
c.2106G>A (p.Met702Ile)	Conflicting interpretations of pathogenicity	criteria provided, conflicting interpretations
c.2113G>T (p.Ala705Ser)	Likely benign	criteria provided, single submitter
c.2113G>C (p.Ala705Pro)	Conflicting interpretations of pathogenicity	criteria provided, conflicting interpretations
c.2120A>T (p.Asp707Val)	Likely pathogenic	criteria provided, multiple submitters, no conflicts
c.2125A>G (p.Arg709Gly)	Likely pathogenic	criteria provided, single submitter
c.2131T>G (p.Cys711Gly)	Pathogenic	no assertion criteria provided
c.2131T>A (p.Cys711Ser)	Likely pathogenic	criteria provided, single submitter
c.2132G>T (p.Cys711Phe)	Likely pathogenic	criteria provided, single submitter
c.2132G>A (p.Cys711Tyr)	Likely pathogenic	criteria provided, multiple submitters, no conflicts
c.2140G>C (p.Glu714Gln)	Likely pathogenic	criteria provided, single submitter
